# Nuclear Pore Proteins Nup153 and Megator Define Transcriptionally Active Regions in the *Drosophila* Genome

**DOI:** 10.1371/journal.pgen.1000846

**Published:** 2010-02-12

**Authors:** Juan M. Vaquerizas, Ritsuko Suyama, Jop Kind, Kota Miura, Nicholas M. Luscombe, Asifa Akhtar

**Affiliations:** 1 European Bioinformatics Institute, Cambridge, United Kingdom; 2 Genome Biology Unit, European Molecular Biology Laboratory, Heidelberg, Germany; 3 Centre for Molecular and Cellular Imaging, European Molecular Biology Laboratory, Heidelberg, Germany; 4 Laboratory of Chromatin Regulation, Max Planck Institute of Immunobiology, Freiburg, Germany; The Babraham Institute, United Kingdom

## Abstract

Transcriptional regulation is one of the most important processes for modulating gene expression. Though much of this control is attributed to transcription factors, histones, and associated enzymes, it is increasingly apparent that the spatial organization of chromosomes within the nucleus has a profound effect on transcriptional activity. Studies in yeast indicate that the nuclear pore complex might promote transcription by recruiting chromatin to the nuclear periphery. In higher eukaryotes, however, it is not known whether such regulation has global significance. Here we establish nucleoporins as a major class of global regulators for gene expression in *Drosophila melanogaster*. Using chromatin-immunoprecipitation combined with microarray hybridisation, we show that Nup153 and Megator (Mtor) bind to 25% of the genome in continuous domains extending 10 kb to 500 kb. These Nucleoporin-Associated Regions (NARs) are dominated by markers for active transcription, including high RNA polymerase II occupancy and histone H4K16 acetylation. RNAi–mediated knock-down of Nup153 alters the expression of ∼5,700 genes, with a pronounced down-regulatory effect within NARs. We find that nucleoporins play a central role in coordinating dosage compensation—an organism-wide process involving the doubling of expression of the male X chromosome. NARs are enriched on the male X chromosome and occupy 75% of this chromosome. Furthermore, Nup153-depletion abolishes the normal function of the male-specific dosage compensation complex. Finally, by extensive 3D imaging, we demonstrate that NARs contribute to gene expression control irrespective of their sub-nuclear localization. Therefore, we suggest that NAR–binding is used for chromosomal organization that enables gene expression control.

## Author Summary

The eukaryotic genome is spatially distributed in a highly organized manner, with chromosomal regions localizing to well-defined sub-nuclear positions. This organization could have a profound effect on chromatin accessibility and transcriptional activity on a genome-wide level. Using high-resolution, genome-wide, chromatin-binding profiles we show that the nuclear pore components Nup153 and Megator bind to quarter of the Drosophila genome in form of chromosomal domains. These domains represent active regions of the genome. Interestingly, comparison of male and female cells revealed enrichment of these domains on the male X chromosome, which represents an exceptionally active chromosome that is under dosage compensation control to equalize gene expression due to differences in X chromosome number between males and females. Based on extensive 3D image analysis, we show that these chromosomal domains are contributed by both peripheral as well as intranuclear pool of these proteins. We suggest that chromosomal organization by nucleoporins could contribute to global gene expression control.

## Introduction

The spatial organisation of DNA, both at the nucleotide and chromosomal levels, allows efficient storage of genetic information inside the nucleus. However, DNA-dependent processes such as transcription, require the chromosomal structure to be modified in order to allow access to this information.

The regulation of chromatin accessibility is an intensely studied subject [1],[2]. Molecular and genomic investigations have examined how nucleotide sequences and ATP-dependent chromatin-remodelling enzymes specify the locations for nucleosomal-binding, and how histone-modifying enzymes modulate the stability of histone-nucleic acid interactions. These enzymes are recruited to precise genomic loci with the aid of sequence-specific DNA-binding transcription factors. In turn, particular histone modifications influence transcription factor-binding to target sites on the genome, so controlling transcriptional initiation. Despite the importance of these cis- and trans-acting factors on the local chromosomal environment and the transcription of nearby genes, it has become increasingly clear that they explain just one level at which chromatin is regulated [3],[4].

The eukaryotic genome is spatially distributed in a highly organised manner, with entire chromosomal regions localising to well-defined sub-nuclear positions [5]. This organisation has a profound effect on chromatin accessibility and transcriptional activity on a genome-wide level [6]–[8]. For instance, chromosomal regions at the nuclear envelope tend to form closed heterochromatin, a structure that is generally indicative of transcriptional repression [9]. Genomic studies in *Drosophila melanogaster* and humans established that lamins—proteins lining the nuclear membrane [10]—are major contributors to sub-nuclear localisation and gene regulation [11],[12]. Comparisons of binding profiles with gene expression data and histone marker information showed that chromosomal regions containing dense lamin-binding were transcriptionally repressed.

Although the nuclear periphery has been primarily associated with repression, recent evidence has also suggested a role for membrane components in transcriptional activation [9], [13]–[16]. The nuclear pore complex is a large structure comprising about 30 protein subunits, and it is the primary channel through which macromolecules traverse the nuclear envelope [17]. Interestingly, investigations in *Saccharomyces cerevisiae* identified subunits of the nuclear pore complex that preferentially bound transcriptionally active genes [18]. Moreover, several target loci such as GAL2 and INO1 were found to relocate from the interior to the periphery upon activation [13], although there were exceptions to this behaviour [19]–[22]. Thus, it is becoming increasingly clear that nuclear periphery components can have both positive and negative influence on gene regulation.

Since there are differences in the composition of the nuclear envelope—such as the lack of lamins—it is important to also study the contribution of nuclear envelope components in gene regulation in higher organisms [9], [17], [23]–[25]. So far just one study has explored the global interactions of nucleoporin subunit Nup93 with human chromosomes 5, 7 and 16 [26]; the publication reported only a low density of binding sites, and their influence on gene regulation was inconclusive.

Recently, we revealed a biochemical association between nucleoporins and the dosage compensation apparatus in higher eukaryotes including humans [27]. In *Drosophila*, the Male Specific Lethal (MSL) complex offsets the imbalance in the number of sex chromosomes in males and females by doubling the expression of genes on the male X chromosome [28],[29]. By purifying enzymatically active MOF complexes, we identified interactions with the nucleoporins Nup153 and Megator (Mtor). Strikingly, depletion of either subunit resulted in the loss of dosage compensation in male cells. Therefore, our work suggested a vital role for nucleoporins in promoting transcriptional activation on a large-scale.

Here, we present the first genome-wide study of nucleoporin-binding in a higher eukaryote. Using chromatin immunoprecipitation followed by hybridisation to high resolution tiling microarrays, we show that Nup153 and Mtor interact with 25% of the Drosophila genome in large domains spanning 10–500 kb in size. These regions—which we term nucleoporin associated regions (NARs)—contain large numbers of highly expressed genes, and are enriched for markers of active transcription including RNA polymerase-binding and histone H4 lysine 16 acetylation. Additionally, we reveal a remarkably high density of NARs on the male X chromosome, which correlate extremely well with the binding pattern of the dosage compensation complex. Finally, we demonstrate that chromosomal regions bound by these nucleoporins are composed of peripheral as well as non-peripheral pools of these proteins but interestingly the X chromosomal target regions are preferentially localised closer to the nuclear periphery. In summary, we firmly establish nucleoporins as a major class of chromatin-binding proteins in higher eukaryotes, with a general role in transcriptional regulation and three-dimensional chromosomal organisation. Finally we show for the first time, the importance of nucleoporin-binding not only as a mechanism for transcriptional control, but also in maintaining a complex organism-level biological system namely dosage compensation.

## Results

### Nup153 and Mtor bind chromatin in a genome-wide fashion

**Figure 1:**
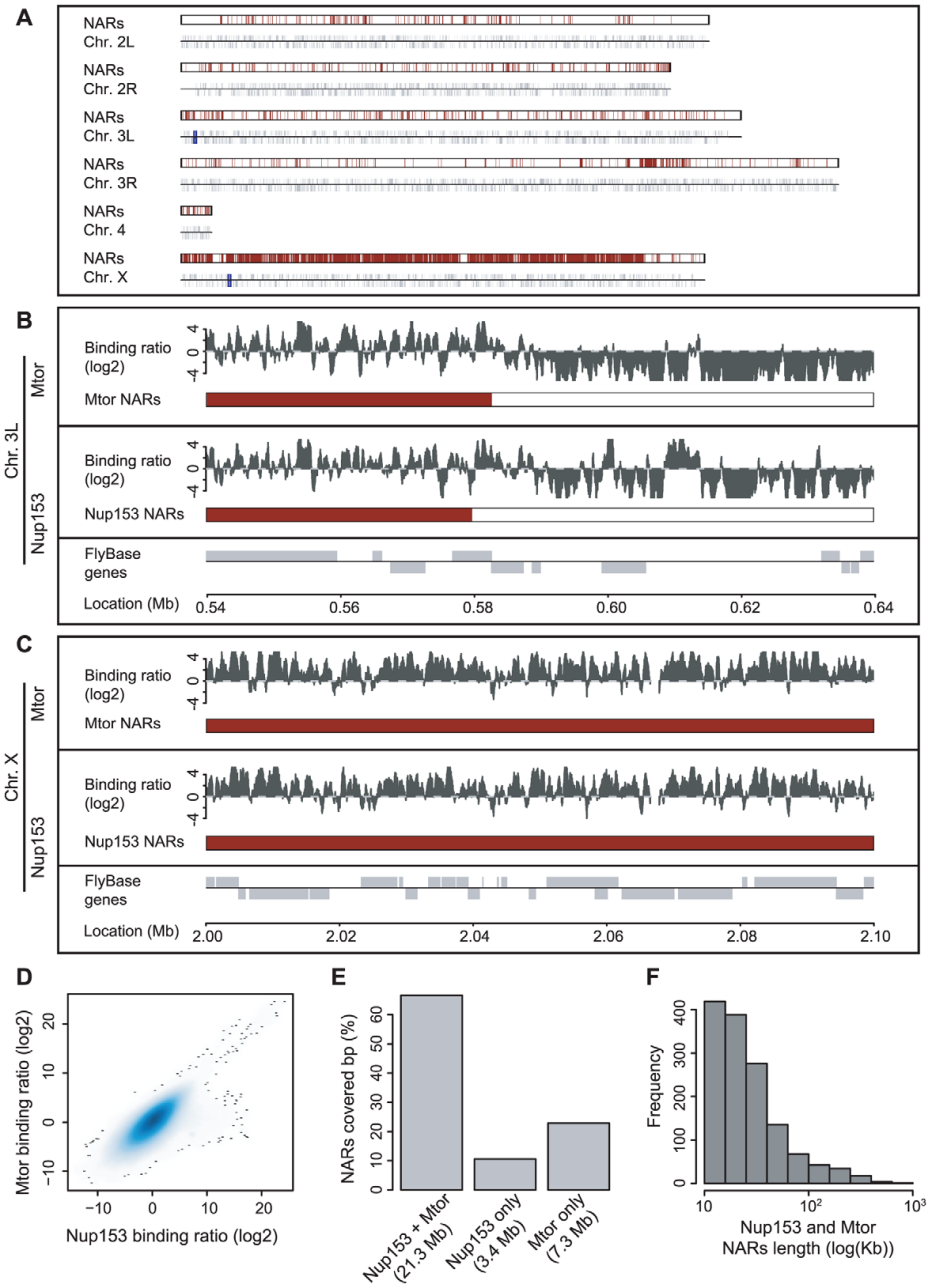
Nup153 and Megator bind the *Drosophila* genome on a large scale. (A) Karyotype representation of the *Drosophila* genome; the upper track depicts the occurrence of high-density nucleoporin-binding in SL-2 cells and the lower track shows the location of annotated genes. Termed Nucleoporin Associated Regions (NARs), high-density binding occurs across 25% of the genome and there is particularly high occupancy on the male X chromosome. (B) Magnified view of Nup153 and Mtor-binding on chromosome 3L. For each nucleoporin, the upper track displays the processed ChIP/input profile and the lower track colours the sections identified as NARs. Note that Nup153 and Mtor show very similar patterns of binding. (C) Magnified view of nucleoporin-binding and NAR occurrence on chromosome X. There is much denser binding on this chromosome compared with autosomes. (D) Smoothed scatter plot displaying the ChIP/input binding ratios for Nup153 and Mtor (*r* = 0.77). (E) Barplot representing the overlap in NARs defined by Nup153 and Mtor binding profiles. (F) Histogram of Nup153 and Mtor NAR length distributions.

We produced DNA-binding profiles for nuclear pore components Mtor and Nup153 in *Drosophila* male SL-2 and female KC cell lines using chromatin immunoprecipitation followed by hybridisation to Affymetrix tiling arrays [30],[31] (Figure 1). Raw data were processed as in Kind et al (2008) to minimise false-positive signals from aberrant array probes (Figure S1) [32].

The ChIP-chip profiles for the two proteins strongly correlate, indicating they bind to similar locations throughout the genome (*r* = 0.77 and 0.88 for SL-2 and KC cells respectively; Figure 1D, Figure S4). We confirmed the reproducibility of results by performing three biological replicates for each condition (*r* = 0.73), and we validated binding at 18 control genes by real-time PCR in triplicate (Figure S2).

Both Mtor and Nup153 exhibit extensive binding across the whole genome, and together they bind to 42% of the *Drosophila* genome (calculated as a fraction of base-pairs covered with two-fold cut-off). Thus nucleoporins represent a new class of global chromatin-binding proteins for higher eukaryotes.

### Nucleoporin-binding occurs in large chromosomal domains

Visual inspection of the ChIP-chip profiles reveals that Nup153 and Mtor interact with the genome in a manner not observed for traditional transcription factors (Figure 1B and 1C) [33]. Instead of associating with discrete loci, nucleoporins bind extended chromosomal regions that alternate between domains of high-density binding with those of low occupancy.

In order to analyse the visual observations in a statistically rigorous fashion, we quantified binding that takes place within a 10 kb sliding window that was scanned along the genome (see Materials and Methods). Windows containing more than 70% binding (as a proportion of array probes with positive binding signal) were classified as Nucleoporin Associated Regions (NARs), and neighbouring windows reaching this threshold were grouped together as continuous NARs. The detection method is robust: the 70% threshold ensures that no NARs are found when binding sites are randomly distributed across the genome and we identify very similar sets of NARs for windows ranging 5 kb to 500 kb in size. Moreover, application of the domain-finding approach described by Guelen et al [11] returns over 80% agreement with our method (in terms of base-pairs classified as NARs).

There is considerable NAR-occurrence (Figure 1A–1C); in male SL-2 cells, a total of 1,384 NARs cover a quarter of the entire *Drosophila* genome (25Mb and 29Mb for Nup153 and Mtor respectively) and in female Kc cells 1,865 NARs occupy a similar proportion of the genome (33Mb and 35Mb for Nup153 and Mtor respectively; Figure S3). Most domains range in size from 10 kb to 100 kb, although some even extend to over 500 kb (Figure 1F, Figure S4). Most nucleotide positions within NARs are occupied by both Nup153 and Mtor. Moreover, even where the overlap is not perfect, NARs tend to occur in similar genomic loci (Figure 1E; Figure 1B chromosomal positions 560,000–600,000). Most importantly, NARs occur in gene-rich areas that encompass over 4,700 protein-coding genes whose activities might be affected by nucleoporin-binding.

### Nucleoporin-binding demarcates actively transcribed chromosomal regions

**Figure 2:**
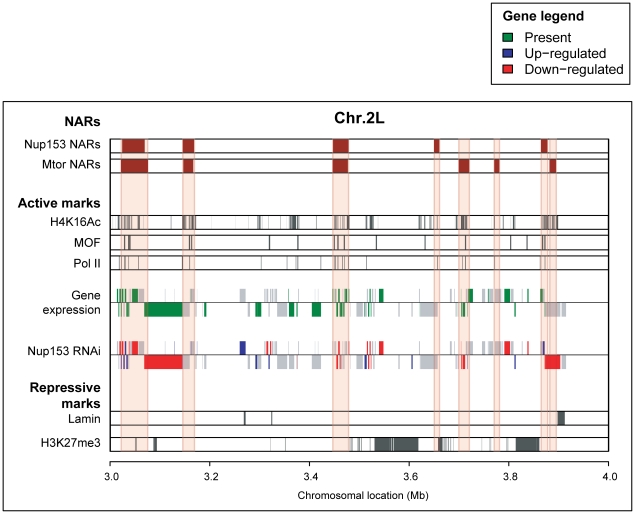
NARs define transcriptionally active regions of the genome. Genome-track view of 1Mb section on chromosome 2L. NARs are enriched for transcribed genes compared with non-NARs (gene expression track; green shading), and a large proportion of genes are down-regulated upon Nup153-depletion (Nup153 RNAi track; red shading). NARs also align with markers of a transcriptionally active chromatin structure (H4K14Ac, MOF and PolII tracks; grey shading), but exclude markers for inactive chromatin (lamin, H3K27me3; grey shading).

A direct relationship between nucleoporin-binding and gene expression has not been established so far in higher eukaryotes. Therefore, we explored the impact of NARs on transcriptional regulation by examining the activity of genes encoded within these regions (Figure 2; Tables S1, S2).

We measured gene expression levels using Affymetrix GeneChips (see Materials and Methods). Using the present-absence calls defined by the MAS5.0 algorithm [34], we detected the expression of 6,478 and 6,219 genes in SL-2 and Kc cells respectively. These genes are preferentially located within NARs: 63% of genes inside NARs are expressed compared with just 40% outside, indicating a significantly elevated transcriptional activity in the former (*p*-value <2.2e^−16^). This observation is supported by data quantifying RNA polymerase II-occupancy (Figure 2; Tables S1, S2); by mapping publicly available ChIP-chip data [35], we find the Pol II-binding is highly enriched at the promoters of genes inside NARs compared with those outside (*p*-value <2.2e^−16^).

Recent publications demonstrated that histone modifications, MOF acetyltransferase- and lamin-binding are robust genome-wide indicators of transcriptional activity. In both SL-2 and KC cells, acetylated histone H4 lysine (H4K16Ac) and MOF-binding [32]—strong markers for active transcription—are extremely prominent within NARs (Figure 2; Tables S1, S2; *p*-value <2.2×10^−16^). In contrast, histone H3 lysine 27 tri-methylation [36] and lamin-binding [12]—markers of transcriptional repression—are enriched outside NARs (Figure 2, Figure S5; Tables S1, S2; *p*-value <2.2e^−16^).

Finally, we confirmed a causal link between nucleoporin-binding and transcriptional regulation by measuring gene expression levels following RNAi-mediated knock-down of Nup153 (Figure 2, Figure S7; Tables S1, S2). The depletion results in large and wide-spread transcriptional changes in cells collected after seven days: 5,684 genes −40% of Drosophila genes represented on the array—are differentially expressed in SL-2 cells (*p*-value <0.05). Moreover, there is a large enrichment of down-regulated genes within NARs (29% of all genes; 40% of ‘present’ genes) compared with non-NARs (19% of all genes; *p*-value <2.2e^−16^). We obtain similar enrichments for cells collected five days after RNAi-treatment, and also upon Mtor-depletion (data not shown). These observations strongly indicate that nucleoporin-binding promotes a high-level of transcriptional activity, which may be due to the formation of an open chromatin environment.

### NARs are enriched on the male X chromosome

One of the most important manifestations of gene expression control in higher eukaryotes is dosage compensation for different number of sex chromosomes between the two sexes. In *Drosophila*—in which females have two X chromosomes but males possess only a single X—the dosage compensation complex offsets the imbalance in gene content by doubling the expression of the male X chromosome. Thus, the chromosome represents an outstanding example of an exceptionally highly transcribed genomic region.

**Figure 3:**
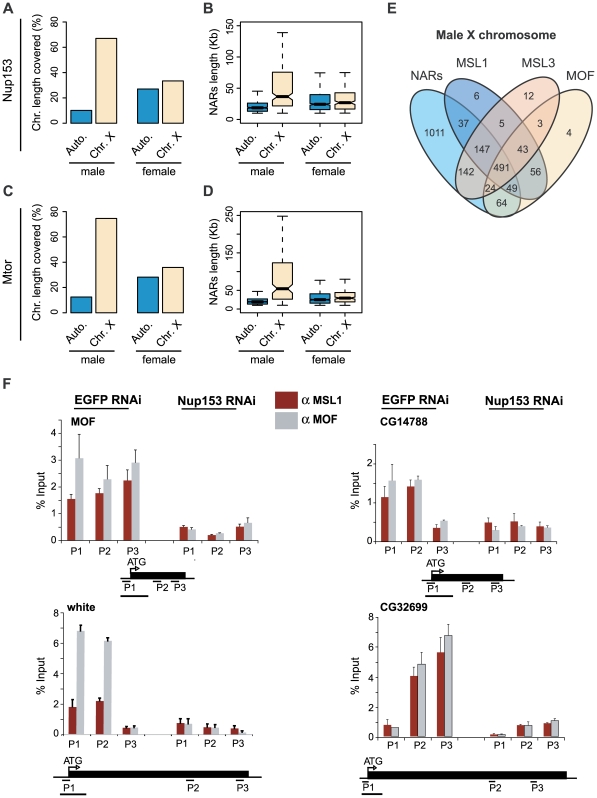
Male X chromosome is especially enriched for NARs. Percentages of NAR occupancy on male and female autosomes and X chromosome for (A) Nup153 and (C) Mtor. In males, NARs are particularly enriched on the X chromosome compared with autosomes, whereas NARs occur evenly throughout in females. NAR length distributions for (B) Nup153 and (D) Mtor. NARs are much longer on the male X chromosome. (E) Overlap between NARs and MSL1-, MSL3- and MOF-binding; numbers represent gene counts. (F) Effect of Nup153-depletion on MSL1- (red shading) and MOF-binding (grey shading) to four X-chromosomal target loci. DNA prepared from cells treated with EGFP (control) or Nup153 dsRNA was immunoprecipitated and analysed by qPCR using primers for the beginning (P1), middle (P2) and end (P3) of genes. Error bars represent the standard deviation in measurements from three replicate experiments. Recovered DNA is shown as a percentage of input DNA.

In order to explore the association of Nup153 and Mtor with the dosage compensation complex further, we compared the patterns of nucleoporin-binding in male SL-2 and female Kc cells (Figure 1A, Figure 3A–3D, Figure S3). There is a dramatic difference between the two sexes: in females, NARs are evenly distributed throughout the entire genome with only a 1.2-fold difference in % NAR occupancy between chromosome X (7.4Mb and 33% for Nup153; 8.0Mb and 36% for Mtor) and autosomes (26.0Mb and 27% for Nup153; 27.1Mb and 28% for Mtor); but in males, NARs are overwhelmingly biased towards the X chromosome (14.9Mb and 67% for Nup153; 16.6Mb and 75% for Mtor) compared with the autosomes (9.7Mb and 10% for Nup153; 12.0Mb and 12% for Mtor) with a 6-fold difference in occupancy. Further, domains on the male X chromosome (median length = 62Kb, 94Kb for Nup153 and Mtor respectively) are much longer than those found on any other chromosomes (median length = 22Kb for Nup153 and Mtor in male autosomes, ∼35Kb for female autosomes and X chromosome).

Having established that the nucleoporins are enriched on the male X chromosome, we explored the association with the dosage compensation system further. Recently, we demonstrated that the members of the dosage compensation complex—MSL1, MSL3 and MOF—preferentially bind to the male X chromosome [32]. A comparison of this previously published dataset with our current analysis shows that NARs on the male X chromosome coincide very well with the binding sites of the dosage compensation complex (Figure 3E).

We also tested the effects of Nup153-depletion on MSL1 and MOF-binding to 10 known target loci using chromatin-immunoprecipitation followed by qPCR. X-chromosomal binding is severely reduced for both proteins (Figure 3F), and the additional binding to autosomal targets is lost for MOF (Figure S8). The effects are clearly specific to Nup153, as depleting another nucleoporin, Nup50 does not influence MSL1 and MOF-localisation and binding (Figure S9; data not shown). Moreover, the observations are not due to an effect on MSL protein concentrations or defects in the RNA export pathway [27]: we previously showed that MSL levels remain unaffected in Nup153 and Mtor-depleted cells; and impairment of the major export pathways through NFX1-depletion does not disrupt the localisation of the MSL complex to the X chromosomes.

### Spatial localisation of NARs versus non–NARs in the nucleus

**Figure 4:**
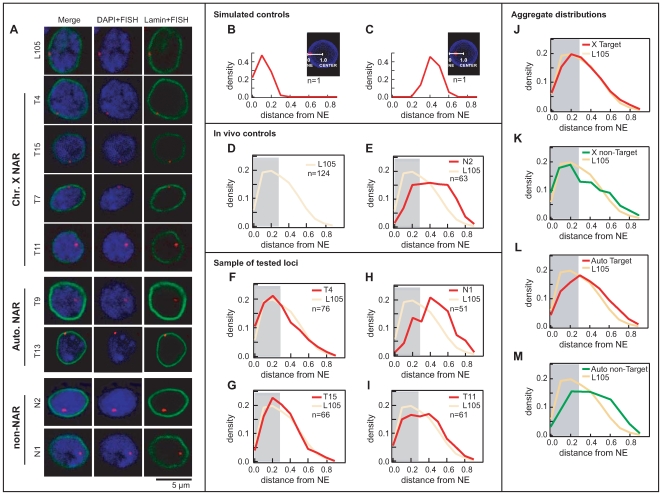
Nup153 and Mtor define NARs both at the periphery and the interior of the nucleus. (A) Representative images of single confocal sections of nuclei containing the FISH signal (red) over DAPI (blue) and immunostained lamin (green). Target genomic regions include a lamin-bound gene (L105), NAR (T4, T15, T7, T11, T9, T13) and non-NAR loci (N1, N2). Probability density plots show the distribution of distance measurements between the FISH signal and the closest point on the nuclear boundary. Simulated nuclei show the ideal distributions for FISH targets located at the (B) periphery and (C) interior. Distances range from 0 at the boundary and 1.0 at the centroid of the nucleus. The grey background represents the theoretical 30% limit for a peripherally localised FISH signal. Observed distributions of *in vivo* controls for (D) peripherally localised L105 and (E) non-peripherally localised N2; the broad spread compared with simulations indicate that the loci display dynamic behaviour in their positioning within the nucleus. (F, G) Predominantly peripheral loci (T4, T15) have distributions that are similar to L105 (shown in yellow), whereas (H, I) predominantly non-peripheral loci (N1, T11) have very different distributions. Aggregate distributions for all NAR targets on (J) the male X chromosome and (L) autosomes, and all non-NAR targets on (K) the male X and (M) autosomes. Targets on the X chromosome are peripherally localised compared with autosomal ones.

Although nucleoporins are primarily located at the nuclear periphery, some display dynamic association with the nuclear pore complex [37], and it remains unclear whether nucleoporin-chromatin interactions would affect transcription at the periphery or within the nucleoplasm. Therefore, we assessed the spatial localisation of different chromosomal regions within the nucleus using three-dimensional imaging of Fluorescence In Situ Hybridisation (3D-FISH) in male and female cells (Figure 4). We selected 26 chromosomal regions of average length 15–20 kb for analysis (Table S3), comprising 18 NAR (targets T1-18) and 8 non-NAR loci (targets N1-8). An independent lamin-bound locus (target L105) was used as a positive control representing a region previously shown to localise at the nuclear periphery [12].

First we checked the localisation of Nup153 and Mtor themselves (Figure S6). Immunostaining of SL-2 cells and salivary glands from male larvae confirm that both proteins predominantly reside in the nuclear periphery, although we also detected some staining within the nucleus. This is consistent with earlier reports that these proteins are dynamic components of the nuclear pore complex, with the capacity to shuttle between different sub-nuclear locations [25],[37].

Next, we used DAPI and lamin protein-immunostaining to assess the nuclear localisation of our target loci. We display a selection of images in Figure 4A: the lamin protein in green defines the nuclear boundary, the DAPI in blue the distribution of genomic DNA, and the FISH signal in red specifies the position of the target locus. In order to account for cell-to-cell variation in localisation that results from the dynamic behaviour of chromatin, we measured the distance between the FISH signal and nuclear boundary for a large number of samples (44<*n*<91). Size differences between nuclei were normalised by representing distances as a percentage of the nuclear radius. In Figure 4B, we show the expected distribution of distances for a simulated locus situated at the periphery; for a FISH signal with 30% radius, we find that most measurements lie between 0% and 30% of the distance to the centre of the nucleus. In contrast simulations for a signal positioned halfway between the periphery and the centre results in a distinct, more symmetrically shaped distribution, with most measurements falling between 20% and 60% of the distance to the centre (Figure 4C; Figures S10, S11; Videos S1, S2, S3, S4).

The lamin-bound L105 locus displays a distribution that is heavily skewed towards the periphery (Figure 4D); however the profile is broader than the simulation, signifying that the locus is present at the interior of the nucleus at least part of the time. On the other hand, target N2 resembles that of the non-peripheral simulation (Figure 4E), albeit with a broader distribution, which indicates that the locus predominantly resides in the interior. Since both loci are NAR-independent, they were assigned as *in vivo* controls representing peripheral and non-peripheral localisation.

Many NAR-target distributions show almost perfect overlap with L105, demonstrating that they are preferentially situated at the periphery (Figure 4F–4G; see Materials and Methods); interestingly however a subset of NAR loci displays distributions that are indicative of non-peripheral localisation (Figure 4I). For non-NARs, targets such as N1 display good overlap with the negative control N2 (Figure 4H), but some are found at the periphery. It is clear, therefore, that many targets regions tested here do not conform to the behaviour expected from NPC-binding.

In fact, we find that NARs from chromosome X tend to reside at the periphery (6 out of 10 targets; Table S4), whereas only a small number of autosomal NARs do so (1 out of 8; Table S4). This is reflected in the aggregate distributions, in which X-chromosomal loci display the characteristic skewed profiles compared with autosomal regions (Figure 4J–4L). Among non-NARs (Figure 4K–4M), autosomal loci are invariably non-peripheral, whereas the X chromosomal targets display a tendency for peripheral localisation; the positioning of the latter is probably influenced by neighbouring NARs as there is such a large amount of binding on the X chromosome. For comparison, peripheral localisation of the X chromosome is absent in female Kc cells (data not shown). Thus in striking contrast to prior expectations, we reveal that interior as well as peripheral populations of nucleoporins bind chromatin and mediate transcriptional activity at NARs. Furthermore, interactions with the X chromosome promotes peripheral localisation of the chromosome—most likely as a result of the overwhelming amount of binding in males—but this is generally not the case for autosomes.

**Figure 5:**
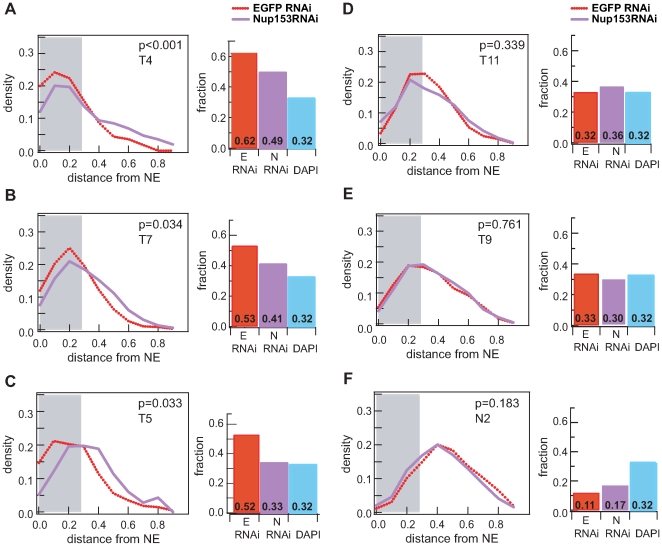
Peripheral localisation is dependent on Nup153. Probability density distributions of distance measurements for mock treated (red) and Nup153-depleted cells (purple). Histograms depict the proportion of nuclei for which the FISH signal is located within the 30% distance threshold (DAPI in blue). (A-C) NAR targets on the male X chromosome (T4, T7, T5) relocalise to the interior upon treatment, indicating that peripheral localisation is dependent on Nup153. (D-F) NAR and non-NAR targets at the interior remain unaffected upon Nup153-depletion.

Finally to confirm the influence of nucleoporins on localisation, we tested the effects of RNAi-mediated Nup153-knockdown for six loci: three peripheral X chromosomal NARs (T4, T5, T7), a non-peripheral X chromosomal NAR (T11), a non-peripheral autosomal NAR (T9) and the non-peripheral control (N2). For each we compared the distribution of Nup153-depleted samples against a mock EGFP RNAi-treatment (Figure 5, Figure S7). All three peripheral targets on the X chromosome displace to a more intra-nuclear position upon loss of Nup153 (Figure 5A–5C; *p*-value <0.05), but in contrast there was no significant change for any of the non-peripheral loci (Figure 5D–5F; *p*-value >0.05). These data suggest that the sub-nuclear positioning of peripheral NARs—specifically those on the male X—depends on the presence of Nup153, whereas the localisation of intra-nuclear loci is independent regardless of whether they are bound by nucleoporins.

## Discussion

The classical view of transcriptional regulation describes the interplay of transcription factors, histones and associated enzymes with DNA in order to recruit the transcriptional machinery to the appropriate genomic loci. However, it has become increasingly clear that these interactions explain only one level at which gene expression is controlled. At a genome-wide level, the spatial organisation of chromosomes within the nucleus is increasingly considered to have a profound effect on chromatin structure and transcriptional activity [5]. In particular, studies in yeast indicate that members of the nuclear pore complex might promote transcription by recruiting chromatin to the nuclear periphery [14],[18]. However, the importance of such regulation in higher eukaryotes has remained unresolved [26].

In this study, we established conclusively that nucleoporins play a central role in mediating transcriptional regulation in a complex, multicellular organism. For the first time in any higher eukaryote, we generated a genome-wide profile of nucleoporin-binding; contrary to preliminary observations, binding is widespread, occurring across 40% of the genome. Thus, we reveal that nucleoporins—Nup153 and Mtor in particular—represent a major new class of global chromatin-binding proteins.

Intriguingly, these proteins interact with the genome differently to traditional transcription factors. Rather than associate with individual loci, nucleoporins bind continuous sections of chromosomes at very high density. Termed NARs, these regions extend up to 500kb in length and occupy 25% of the entire *Drosophila* genome. Moreover, NARs are functionally important as they demarcate regions of open chromatin and transcriptional activity, which is lost on depletion of Nup153. It is significant that the male X chromosome—a prime example for hyper-transcription—is almost entirely occupied by NARs. Therefore, we suggest that Nup153 and Mtor may stimulate transcription by promoting the formation of an open chromatin environment.

In dramatic contrast to expectations, nucleoporin-binding does not automatically lead to localisation at the nuclear periphery, though the male X chromosome is an exception in this regard. Since Nup153 and Mtor are known to be dynamic components of the nuclear pore complex, it appears likely that both peripheral and intra-nuclear pools of nucleoporins contribute to chromatin-binding. Given the dynamic nature of chromatin-localisation, it is also possible that NARs are located at the periphery in a very transient manner, and further developments in imaging techniques will help clarify this. Where NAR-formation and peripheral localisation do coincide however, Nup153 is necessary for sustained positioning.

Chromosomal domains have been implicated in the formation of three-dimensional sub-nuclear structures to coordinate the expression of otherwise distant loci [38] such as the human beta-globin genes [39],[40]. We speculate that NARs may indicate the genomic regions required for the assembly of these transcription factories on a very large scale. Within this context, the dynamic nature of Nup153 and Mtor is significant, as re-localisation of these proteins might allow a basis for global transcriptional control in response to cellular cues. Additionally, given the primary function of the nuclear pore complex in transporting macromolecules to and from the nucleus, Nup153 and Mtor may provide a means to couple transcriptional control with post-transcriptional events. We stress however that the mechanisms behind such processes are the subject of intense research activity and many controversies remain.

Finally, the special link with dosage compensation confirms the importance of nucleoporin-binding not only as a molecular mechanism for transcriptional control, but also in maintaining a complex, organism-level biological system.

## Materials and Methods

### ChIP–chip and qPCR analysis

Chromatin immunoprecipitation combined with microarray hybridisation (ChIP-chip), and qPCR experiments were performed as described previously in Kind et al [32]. Primer sequences are provided in Text S1.

Numerical data from Affymetrix *Drosophila* Tiling 2.0R Arrays (Dm35b_MR_v02) were processed as in Kind et al [32]. Briefly, array data were background corrected using GCRMA and quantile normalised [41]. Log2 (ChIP/input) ratios were calculated using the average from three replicate experiments. Log2 ratios were then smoothed by averaging the signal within a 500 bp window centred on each probe (Figure S1).

### Identification of Nucleoporin Associated Regions (NARs)

Chromosomal regions with high densities of Nup153- and Mtor-binding were identified by sliding a 10 kb window along each chromosome, centred on the start position of each probe. NARs were defined as continuous chromosomal regions containing positive binding signal (ie, log2 ratio >0) for more than 70% of probes. We also implemented the two-stage domain-finding method described by Guelen et al [11]. Our method recovered at least 80% of all probes defined as domains by the Guelen approach.

### RNAi on cultured cells

Nup153 and Nup50 were depleted as previously described in Mendjan et al [27]. Briefly, cells were incubated with dsRNA for five or seven days with a boost on day two. Cells were subsequently harvested for Western blot analysis, ChIP, gene expression profiling, or immunofluorescence experiments. Control experiments were performed using mock treatment (EGFP RNAi).

### Gene expression profiling

Gene expression was measured using Affymetrix *Drosophila*2 GeneChips in triplicate for each condition. Data analysis was performed using publicly available packages in the BioConductor Software Suite [43]. Raw .CEL files were processed using RMA [44] and probe-sets were mapped to genes using the annotation from the Ensembl database (v41) [45].

In control (EGFP-treated) cells, expressed genes were identified as those outputting MAS5.0 ‘present’ cells in all three replicates [34]. For comparisons of Nup153-depleted and mock-treated cells, differentially expressed genes were determined using the Limma package [46]; *p*-values were corrected for multiple-testing using FDR [47] and a significance threshold of *p*-value<0.05 was selected.

### Overlap of NARs with markers for transcriptional activity

We compared the overlap between NARs and genomic features. For ease of comparison, all data were mapped onto the *Drosophila* genome provided by the Ensembl database (v. 41) [45]. Accompanying each entry is the statistical significance of the difference in the amount of genomic feature found within NARs and non-NARs.

(i) Histone H4 lysine K16 acetylation (H4K16Ac; p<2.2e^−16^; t-test): processed ChIP-chip profiles obtained from Kind et al [32]. (ii) MOF-binding (p<2.2e^−16^; Fisher test): processed ChIP-chip profiles obtained from Kind et al [32]. (iii) RNA PolII-occupancy (p<2.2e^−16^; Fisher test): PolII-bound genes obtained from Muse et al [35]. For visualisation purposes in Figure 2, bound genes were represented as 1kb windows centred on the transcription start site. (iv) Gene density (*p*-value <2.2e^−16^; Wilcoxon test): number of genes as annotated by the Ensembl database within a 20kb sliding window with a 1 kb offset. (v) Expressed genes (*p*-value <2.2e^−16^; Fisher test): gene expression measured using Affymetrix *Drosophila2* GeneChips as described above. (vi) Down-regulated genes upon Nup153-depletion (*p*-value <2.2e^−16^; Fisher test): differentially expressed genes in RNAi-treated cells compared with untreated cells as described above. (vii) Lamin-binding (*p*-value <2.2e^−16^; Fisher test): processed ChIP-chip data were obtained from Pickersgill et al [12]. Note that the study used low-resolution cDNA arrays, and therefore unlike the human study, the authors were unable to detect high-density lamin-associated domains. (viii) Histone H3 lysine 27 tri-methylation (H3K27me3; *p*-value <2.2e^−16^; Fisher test): processed ChIP-chip profiles obtained from Schwartz et al [36].

### Fluorescent In Situ Hybridisation on cultured cells

DNA FISH on SL-2 cells was performed as previously described by Lanzuolo et al [48]. Briefly for DNA FISH 1×10^6^ cells were centrifuged, re-suspended in 0.4 ml of medium and placed for 30 min at room temperature on a poly-lysine-coated slide (10 mm diameter). After rinsing with PBS, the cells were fixed with 4% paraformaldehyde in PBT (PBS, 0.1% Tween 20) for 10 min at room temperature. Cells were then washed three times with PBT, incubated for 1 h at room temperature with RNAse A (100 µg/ml in PBT). After rinsing with PBS, cells were incubated with 0.5% Triton in PBS for 10 min at room temperature. Cells were rinsed again with PBS and incubated with 20% glycerol in PBS for 30 min at room temperature. Cells were then frozen in liquid nitrogen, thawed at room temperature and soaked in 20% glycerol in PBS, repeatedly four times. After washing the cells again with PBS three times, they were incubated for 5 min in 0.1N HCl, briefly rinsed in 2XSSC twice, and stored in 50% formamide, 2XSSC, 10% dextransulphate, pH 7.0. Fluorescent probes were prepared with the FISH Tag DNA Kit (Invitrogen, Carlsbad, CA), dissolved in the hybridization mixture (50% deionized formamide, 2XSSC, 10% dextransulphate, salmon sperm DNA at 0.5 mg/ml), applied to cells and sealed under coverslips with rubber cement. Probe and cellular DNA were denatured simultaneously on a hot block at 78°C for 3 min. Hybridization was carried out in a humid atmosphere at 37°C for 1 d. After hybridization, slides were washed in 2XSSC three times for 5 min at 37°C, and in 0.1XSSC three times for 5 min at 45°C, rinsed in PBS twice and counter-stained with DAPI.

For immuno-FISH, the following procedure is added after washing with 0.1XSSC at 45°C. Wash twice with 2XSSC 5 min each at RT. Blocking with (TNT buffer; 0.1M Tris-HCl pH 7.5, 0.15M NaCl, 5% BSA) for 1 h at RT. Anti-lamin antibody is incubated for overnight at 4°C in TNT buffer, wash with wash buffer three times for 5 min. Second antibody is applied in TNT buffer for 2–3 h at RT, wash with wash buffer (0.1M Tris-HCl pH 7.5, 0.15M NaCl), including DAPI staining as described above. Cells were mounted on the glass slide with FluoromountG (Southern Biotech. Birmingham, AL). Three-dimensional image stacks were taken with Leica SP5 confocal microscope (Leica Microsystems, Exton, PA) using an x63 oil immersion objective with a numerical aperture of 1.4, and zoom 3.2±0.2.

To perform DNA FISH on target and non-target probes, approximately 15 kb region were chosen, except for the repeated sequence, in the genome and amplified by PCR from genomic DNA with 5–10 primers pairs, each covering around 0.5–3 kb. Primer sequences are available on request.

### Image analysis of FISH localisation

To determine quantitatively the three-dimensional position of the FISH signal within the nucleus, we used the ImageJ software [49]. The nuclear envelope was initially defined by segmentation of the DAPI image using the automated Otsu thresholding algorithm. The boundary definition was then refined against the lamin-staining, flagging significant deviations between the two signals if necessary. Figure S10 shows a schematic diagram of the procedure. We also display a distribution of radii calculated for 62 nuclei, demonstrating that the DAPI and lamin signals provide very consistent definitions of the nuclear boundary. Segmented images were then stacked in order to recreate the three-dimensional nucleus.

Next we calculated the distances between the FISH signal and the nuclear boundary (Figure S10, S11, Videos S1, S2, S3, S4). The segmented three-dimensional images of the nucleus were converted into a three-dimensional distance map using the Local Thickness plug-in (http://www.optinav.com/Local_Thickness.htm). We thresholded the FISH images to identify voxels within the nucleus that corresponded to the FISH signal and we measured the distances between all such voxels and the closest point on the nuclear boundary. For each nucleus we calculated the mean distance, and then for each test locus, we use the set of mean distances for all nuclei to plot the distance distribution. Similar results were obtained when we used the centre of mass of the FISH signal as the reference point instead of the mean distances for individual voxels (data not shown).

In total, we examined 1,712 nuclei (35–91 samples for each target locus; total 1,172 nuclei for NAR; total 540 nuclei for non-NARs). For a given target, we compiled all distance measurements from all relevant nuclei to produce a distribution of distances as shown in Figure 4 and Table S4.

The lamin L105 and N2 non-NAR targets were selected as *in vivo* controls with representative distributions for peripheral and non-peripheral sub-nuclear localisation. We compared the localisation of each target locus by comparing its distance measurements against the L105 and N2 controls separately. Statistical significance was calculated using the Wilcoxon test, with a FDR-corrected threshold of *p *<0.05. Briefly, a non-significant *p-*value (ie *p*-value >0.05) compared with the L105 distribution is indicative of peripheral localisation, whereas a non-significant *p*-value (i.e. *p*-value >0.05) compared with the N2 distribution is indicative of non-peripheral localisation.

### Accession numbers

Microarray data are available in the ArrayExpress databaset [42] under accession numbers E-MEXP-2523 (gene expression data) and E-MEXP-2525 (ChIP-chip data).

## Supporting Information

Figure S1 Processing of ChIP-chip data and NAR determination for Nup153. All ChIP-chip assays were performed in triplicate. Raw data were GCRMA-normalised. Triplicates were averaged and binding ratios were calculated relative to average intensities from triplicates of 10% input DNA. Data were then smoothened by using averaging of intensities within a 500bp sliding window centred on each probe. We then calculated the density of positively probes in 10 Kb windows centred on each probe, and used a cut-off of 70% to determine Nucleoporin Associated Regions (NARs). Profiles of the different analysis steps are illustrated for a 200 Kb region of chromosome X in SL-2 cells: GCRMA-normalised intensities for individual probes across three biological replicates (light orange); mean intensity values of the three biological replicates for Nup153 binding (orange); GCRMA-normalised intensities and mean values for the input DNA control (light and dark grey); ratios of Nup153-binding and control mean intensity signals (light blue); smoothed ratios using a 500-bp sliding window centred on each probe (dark blue); density of positively bound probes in 10 Kb windows centred on each probe (solid black line) and 70% threshold for detection of NARs (dotted red line); Nup153 NARs (dark red boxes); FlyBase genes in the forward and reverse strand are represented in light grey; coordinates represent the position on the corresponding chromosome. A similar procedure was used to determine NARs in male and female samples for Nup153 and Mtor.
(0.6 MB PDF)

Figure S2 Validation of Nup153 and Mtor target and non-target genes by ChIP-QPCR. Chromatin prepared from SL-2 cells was used for immunoprecipitation using Nup153 (blue) and Mtor (grey) antibodies. Recovered DNA (% Input) was analysed by Q-PCR using primers in the beginning (P1), middle (P2) and end (P3) of genes as shown. Error bars represent standard deviation obtained from three independent experiments.
(0.04 MB PDF)

Figure S3 Nup153 and Mtor NARs in Kc cells. (A) Karyotype representation of Nup153 and Mtor NARs across the genome in Kc cells. (B) Magnified view of a 1Mb region of chromosomal arm 2L. Tracks represent the smoothened binding ChIP/input ratio for Nup153 and Mtor (dark grey), the density of positively bound probes calculated in 10 Kb windows centred on each probe (solid grey line), and NARs (red boxes), for regions with a density of positively bound probes above 70%. (C) Magnified view of a 100 kb region in (B).
(2.11 MB PDF)

Figure S4 Correlation between Nup153 and Mtor binding in Kc cells. (A) Smoothed scatter plot displaying the ChIP/input binding ratios for Nup153 and Mtor (Pearson r = 0.88). (B) Bar chart representing the overlap in NARs defined by Nup153 and Mtor binding profiles. (C) Histogram of Nup153 and Mtor NAR length distributions.
(0.56 MB PDF)

Figure S5 H4K16Ac and H3K27me3 are mutually exclusive throughout the genome. (A) Detail view of H3K27me3 and H4K16Ac modifications in a 1 Mb region of chromosome X in SL-2 cells. H3K27me3 data were obtained from Schwartz et al (2006) [36] and H4K16Ac data were obtained from Kind et al (2008) [32]. For each modification, we used the cut-offs from the original publications to define significant signals. (B) Smoothed scatter plot of H4K16Ac and H3K27me3 modification intensity values. Only data points with significant intensity values are shown. Plot areas with high data density are shown in dark red; plot areas with low are density are shown in dark blue.
(1.39 MB PDF)

Figure S6 Immunostaining of Nup153 and Mtor in salivary glands. Immunostaining of Nup153 and Mtor in salivary glands isolated from 3rd instar male larvae. Salivary glands were co-immunostained with either MSL1 antibody or pre-immune serum (Pre-Mtor, Pre-Nup153) and serum (Mtor and Nup153). Both Nup153 and Mtor show predominantly nuclear rim staining but there is also some diffuse staining within the nucleus. X chromosomal territory is observed with MSL1 staining.
(0.23 MB PDF)

Figure S7 RNAi-mediated depletion of Nup153 in SL-2 cells. (A) Whole extracts were obtained from cells treated with EGFP or Nup153 dsRNA for 0, 3, 5, or 7 days, and separated on SDS PAGE followed by western blot analysis using Nup153 and Tubulin antibodies. Size markers (kDa) are indicated on the right side. (B) Cells treated with EGFP or Nup153 dsRNA were used for immunofluorescence confocal microscopy. Nup153, Nup50, and Lamin antibodies were used for triple-immunostaining and pseudo colours were added using the ImageJ software. A similar strategy was used for MOF, MSL1 and Lamin triple immunostaining. Arrows indicate residual MSL1- or MOF-staining in Nup153-depleted cells.
(0.75 MB PDF)

Figure S8 MOF-binding to autosomal promoters is affected in Nup153-depleted cells. Chromatin prepared from cells treated with EGFP (black) or Nup153 (grey) dsRNA was used for immunoprecipitation using MOF antibody. MOF-binding was scored on six autosomal target promoters. Recovered DNA was analysed by qPCR and is shown as percentage of input DNA (% Input). Error bars represent standard deviations obtained from three independent experiments.
(0.33 MB PDF)

Figure S9 Control RNAi-mediated depletion of Nup50 in SL-2 cells. (A) Whole cells extracts were made from cells treated with EGFP or Nup50 dsRNA for 0, 5, or 7 days, and separated on SDS PAGE followed by western blot analysis using Nup50 and Tubulin antibodies. Size markers (kDa) are indicated on the right side. (B) Cells treated with EGFP or Nup153 dsRNA were used for immunofluorescence confocal microscopy using antibodies against Nup153, Nup50, Mtor, MOF, and MSL1 (shown in red). Nup153, Mtor antibodies, and Hoechst were used for triple-immunostaining and pseudo-colours were added using the ImageJ software. A similar strategy was used for MOF, MSL1, and Lamin triple immunostaining.
(0.43 MB PDF)

Figure S10 Segmentation of DAPI image. (Top left) Nucleus labelled with lamin and stained with DAPI were used for the development of segmentation method (green = lamin, blue = DAPI). (Top centre) Lamin signal was thresholded and then reduced to single pixel rim (green). Detected rim was overlaid to the original lamin image. (Top right) The segmentation strategy were verified by measuring the deviation between DAPI segmented image (black/white) and the lamin rim (green). Pink lines show how these deviations were measured. (Bottom) Probability density distributions of the mean radii of 62 individual nuclei calculated using the DAPI or lamin signals. The median radius for the DAPI segmented edge was 2.45±0.33 µm and for the lamin signal was 2.41±0.35 µm.
(0.05 MB PDF)

Figure S11 Measurement of three-dimensional distance of FISH signals from the nuclear periphery. Three-dimensional brightest-point projection images of a simulated nucleus showing (A) peripheral localisation and (B) non-peripheral localisation. Outlines of nuclear periphery in each z-slice (blue contours, DAPI channel) and FISH signal (red, FISH channel) are shown. Nucleus is rotated on the x-axis with 30 degree increments from top-left to bottom-right panel. Three-dimensional brightest-point projection images of real nuclei with NAR locus (C) T4 and (D) control locus N2. Bar = 5 µm. See also Videos S1-S4.
(0.55 MB PDF)

Table S1 Enrichment of active and repressive markers in NARs and non-NARs in SL-2 and Kc cells.
(0.05 MB PDF)

Table S2 Enrichment of H4K16Ac and gene density in NARs versus non-NARs for SL-2 and Kc cells.
(0.05 MB PDF)

Table S3 Target (T) and non-target (N) regions used for FISH analysis. Start and end show the chromosomal localization coordinates according to release 3 of the *Drosophila melanogaster* genome (R5.11). Genes in each probe set are also indicated. Individual genes within these regions, which were further tested by Q-PCR in this study, are indicated in red.
(0.08 MB PDF)

Table S4 This table accompanies Figure 4. Chromosomal location of the target and non-target regions is indicated. Total number of pixels and nuclei counted is also indicated as well as the statistical significance of each target or non-target region shown separately as well as average of each category.
(0.06 MB PDF)

Text S1 Primer sequences for quantitative PCR; primer sequences for RNAi.
(0.09 MB PDF)

Video S1 3D projection movie of a simulated nucleus with FISH signal at nuclear periphery. Nuclear envelope is shown as blue contours and FISH signal is shown in red. Montages of the movies are shown in Figure S11.
(0.73 MB MOV)

Video S2 3D projection movie of a simulated nucleus with FISH signal located between the periphery and nuclear centre.
(0.73 MB MOV)

Video S3 3D projection movie of real nucleus with NAR locus T4 localised to the periphery.
(0.62 MB MOV)

Video S4 3D projection movie of real nucleus with control locus N2 localised at the interior.
(0.83 MB MOV)
