# Supplementary figures and images for: Nuclear Pore Proteins Nup153 and Megator Define Transcriptionally Active Regions in the Drosophila Genome
Source: PLoS Genet. 2010 Feb 12;6(2):e1000846. doi: 10.1371/journal.pgen.1000846 (PMC2820533; doi:10.1371/journal.pgen.1000846)

Supplementary Figure 1

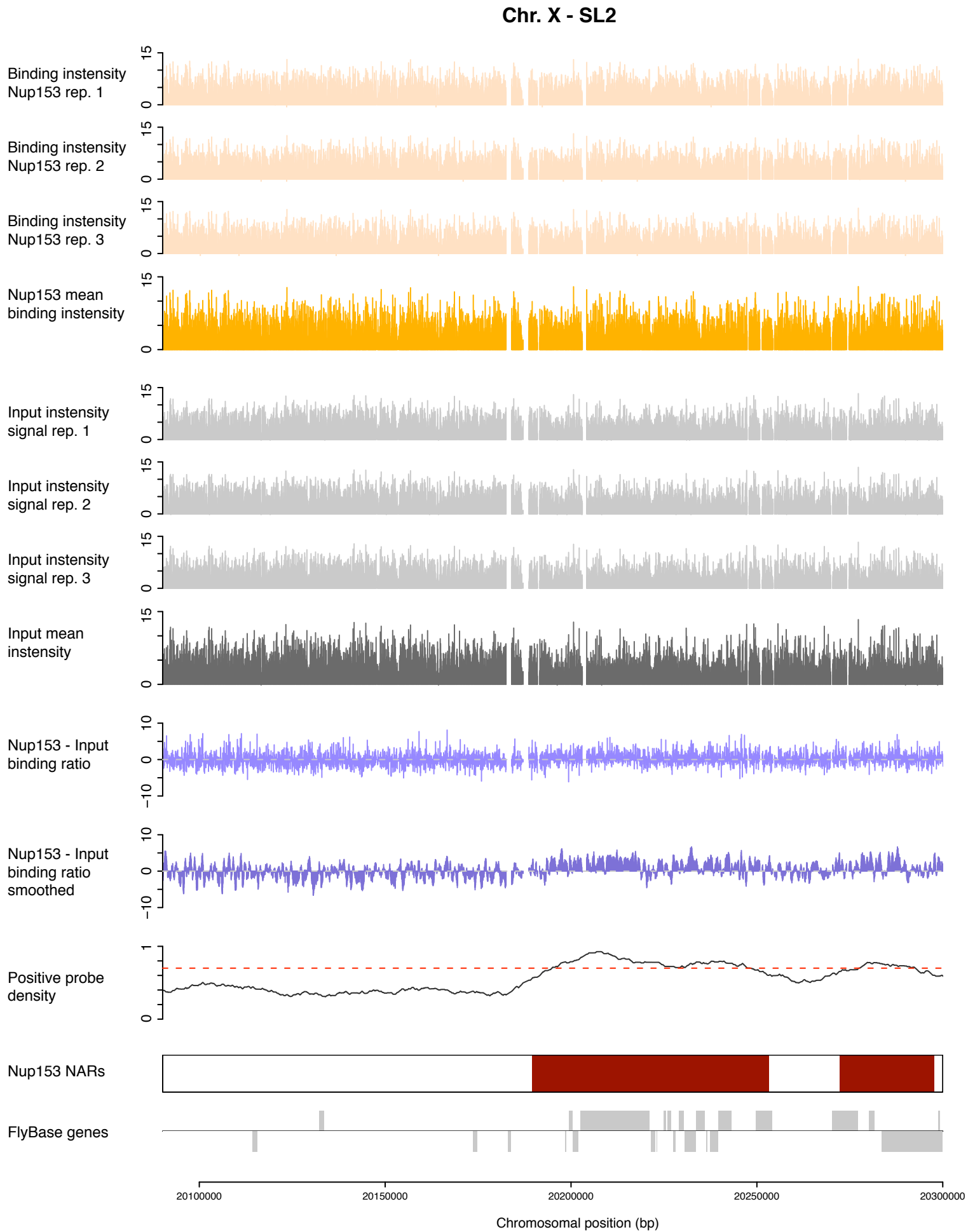

Supplement: Figure S1 — Processing of ChIP-chip data and NAR determination for Nup153. All ChIP-chip assays were performed in triplicate. Raw data were GCRMA-normalised. Triplicates were averaged and binding ratios were calculated relative to average intensities from triplicates of 10% input DNA. Data were then smoothened by using averaging of intensities within a 500bp sliding window centred on each probe. We then calculated the density of positively probes in 10 Kb windows centred on each probe, and used a cut-off of 70% to determine Nucleoporin Associated Regions (NARs). Profiles of the different analysis steps are illustrated for a 200 Kb region of chromosome X in SL-2 cells: GCRMA-normalised intensities for individual probes across three biological replicates (light orange); mean intensity values of the three biological replicates for Nup153 binding (orange); GCRMA-normalised intensities and mean values for the input DNA control (light and dark grey); ratios of Nup153-binding and control mean intensity signals (light blue); smoothed ratios using a 500-bp sliding window centred on each probe (dark blue); density of positively bound probes in 10 Kb windows centred on each probe (solid black line) and 70% threshold for detection of NARs (dotted red line); Nup153 NARs (dark red boxes); FlyBase genes in the forward and reverse strand are represented in light grey; coordinates represent the position on the corresponding chromosome. A similar procedure was used to determine NARs in male and female samples for Nup153 and Mtor. (0.6 MB PDF) [file pgen.1000846.s001.pdf]

**Supplementary Figure 2**

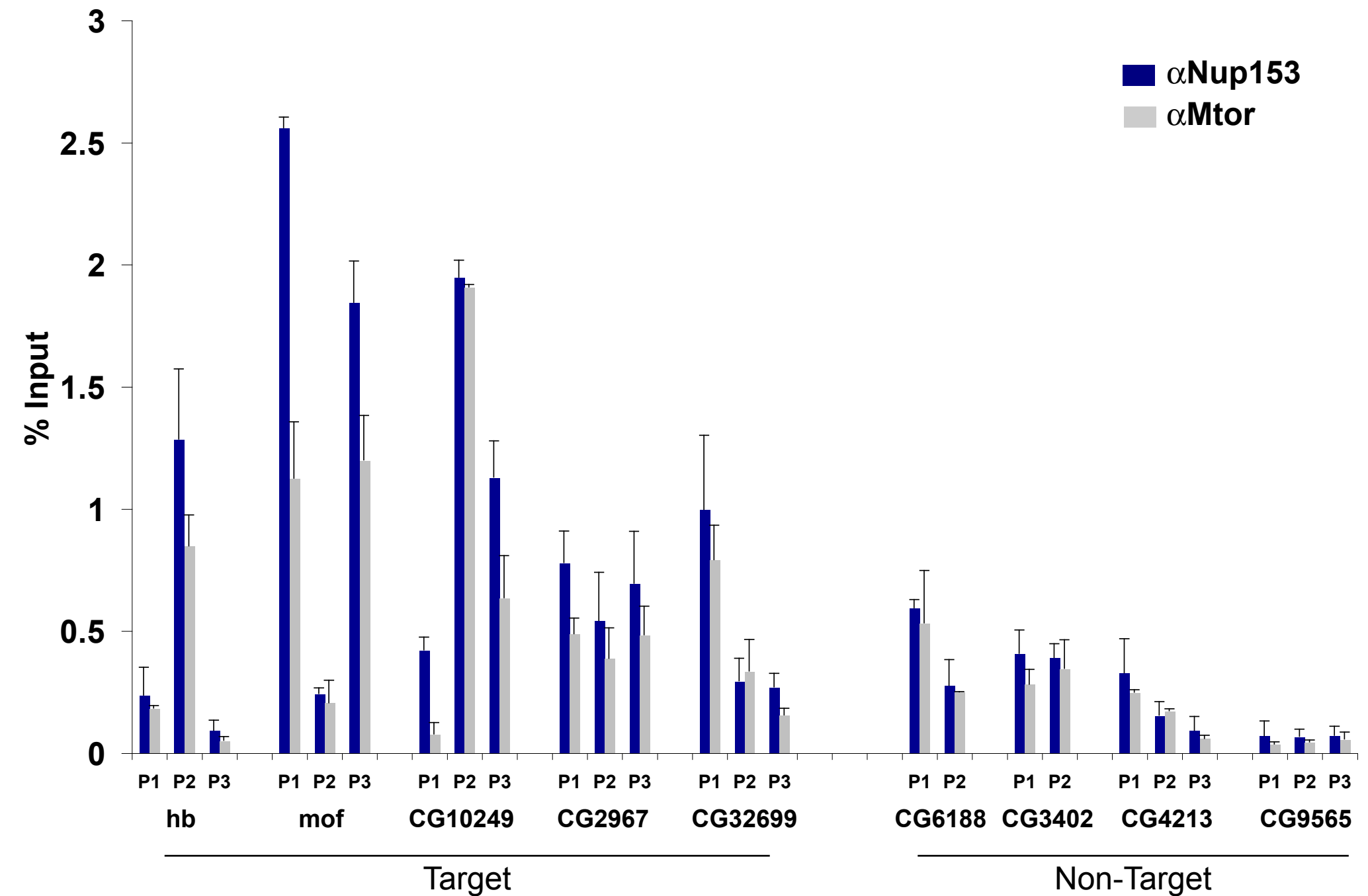

Supplement: Figure S2 — Validation of Nup153 and Mtor target and non-target genes by ChIP-QPCR. Chromatin prepared from SL-2 cells was used for immunoprecipitation using Nup153 (blue) and Mtor (grey) antibodies. Recovered DNA (% Input) was analysed by Q-PCR using primers in the beginning (P1), middle (P2) and end (P3) of genes as shown. Error bars represent standard deviation obtained from three independent experiments. (0.04 MB PDF) [file pgen.1000846.s002.pdf]

Supplementary Figure 3

A

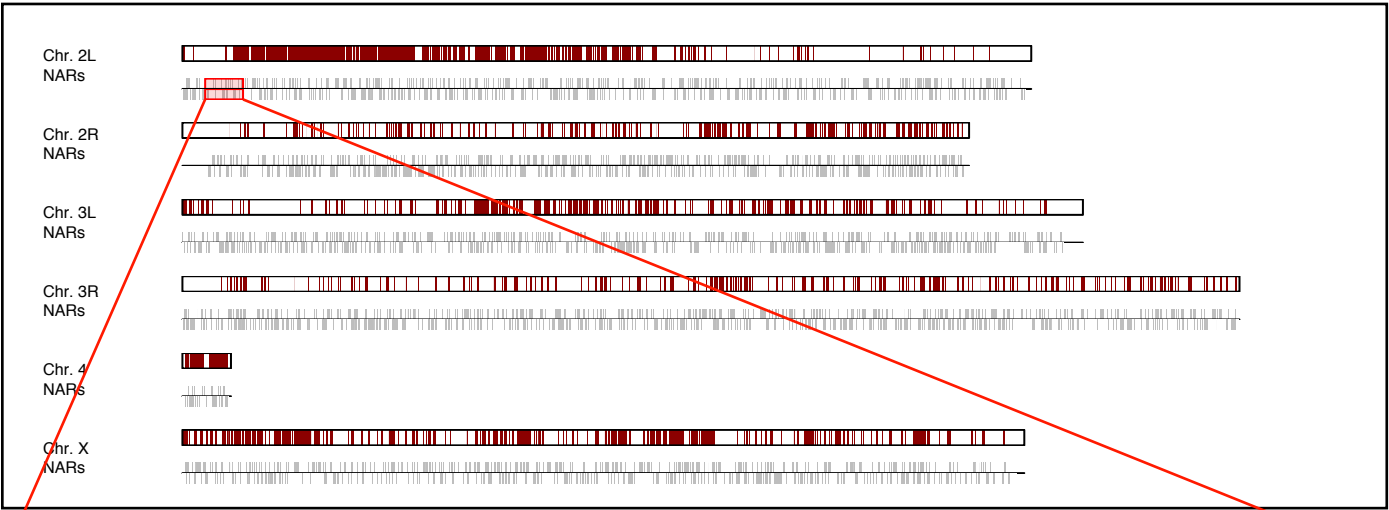

B

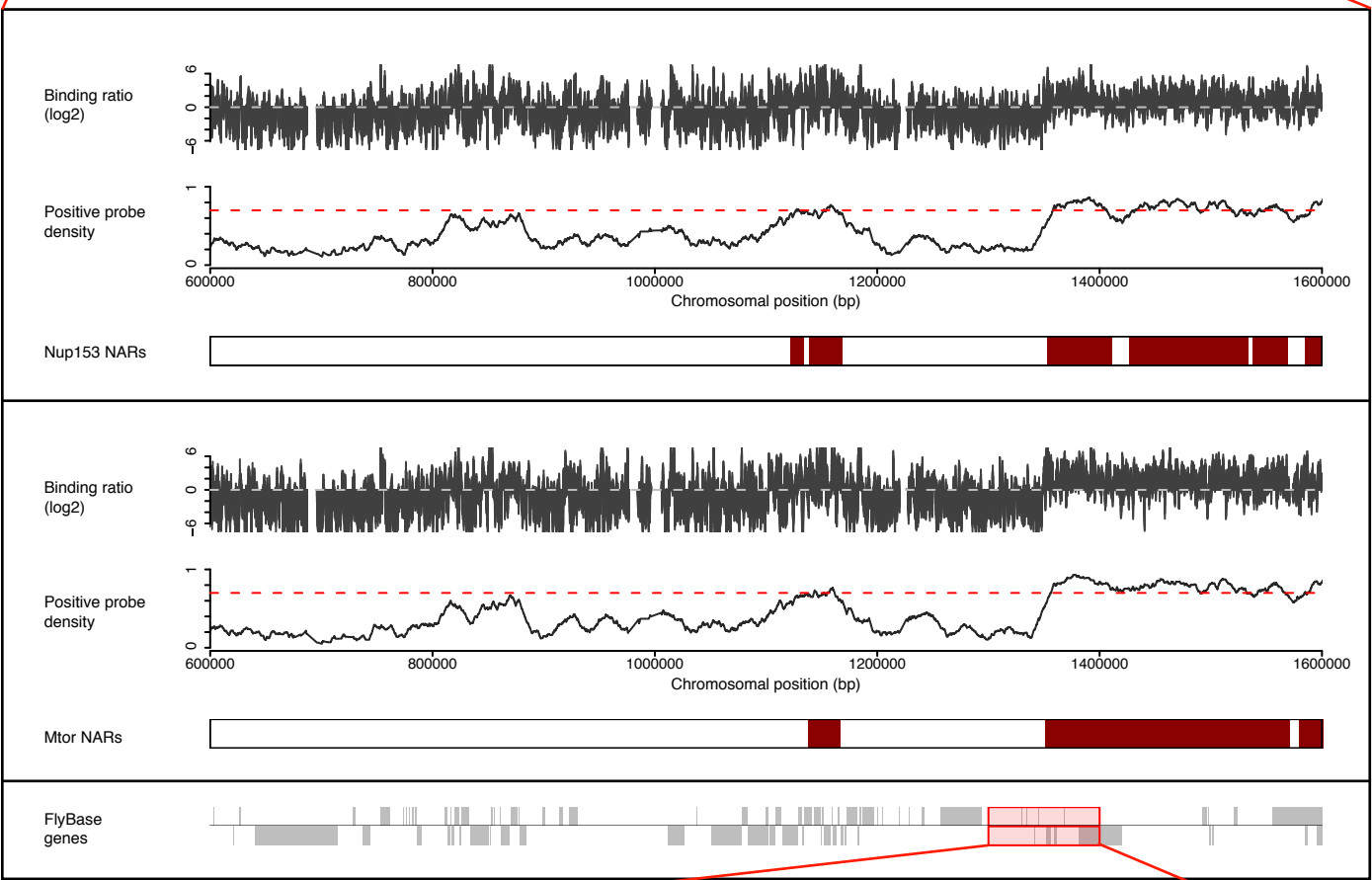

C

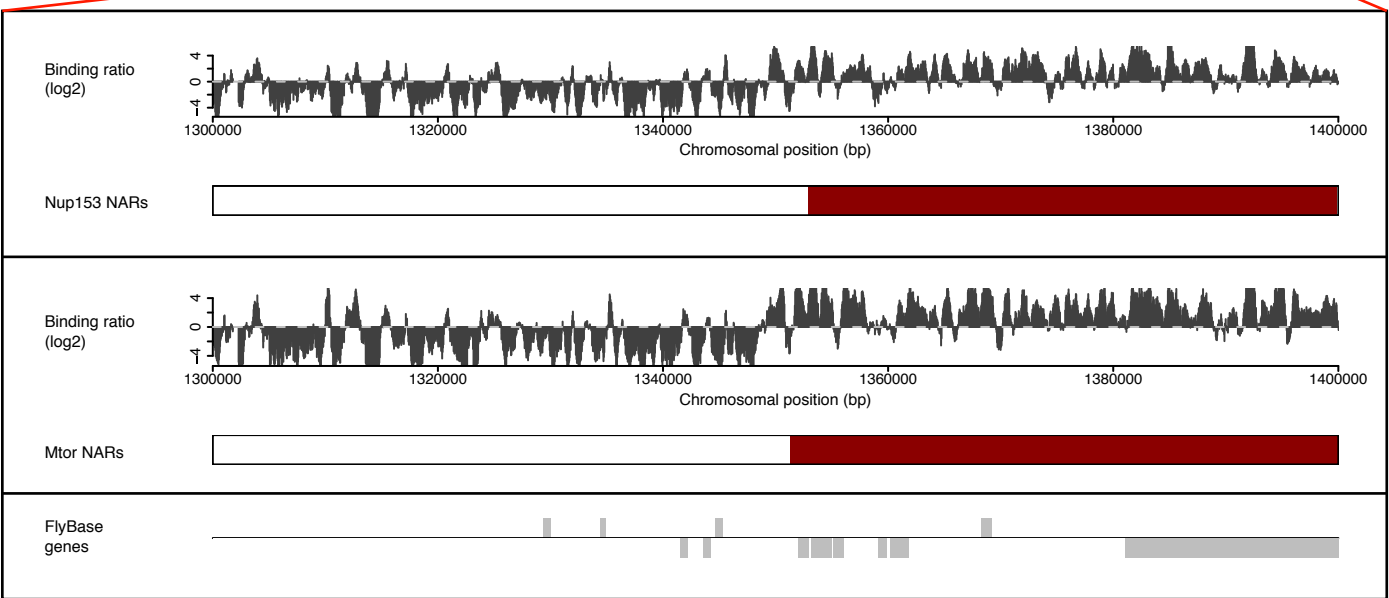

Supplement: Figure S3 — Nup153 and Mtor NARs in Kc cells. (A) Karyotype representation of Nup153 and Mtor NARs across the genome in Kc cells. (B) Magnified view of a 1Mb region of chromosomal arm 2L. Tracks represent the smoothened binding ChIP/input ratio for Nup153 and Mtor (dark grey), the density of positively bound probes calculated in 10 Kb windows centred on each probe (solid grey line), and NARs (red boxes), for regions with a density of positively bound probes above 70%. (C) Magnified view of a 100 kb region in (B). (2.11 MB PDF) [file pgen.1000846.s003.pdf]

# Supplementary Figure 4

**A**

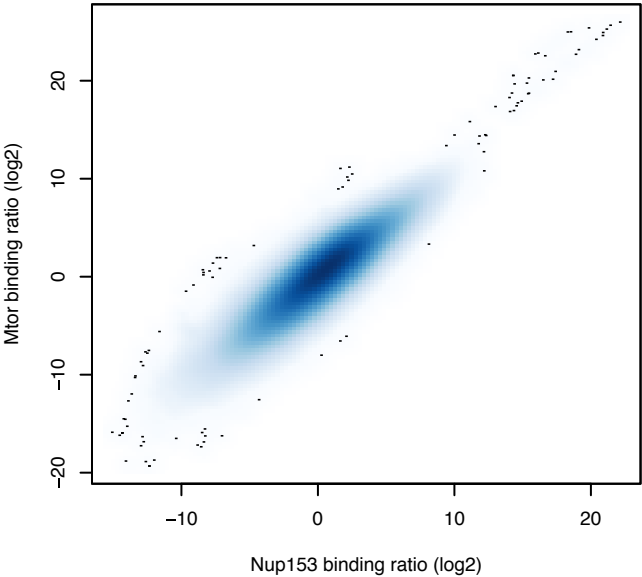

**B**

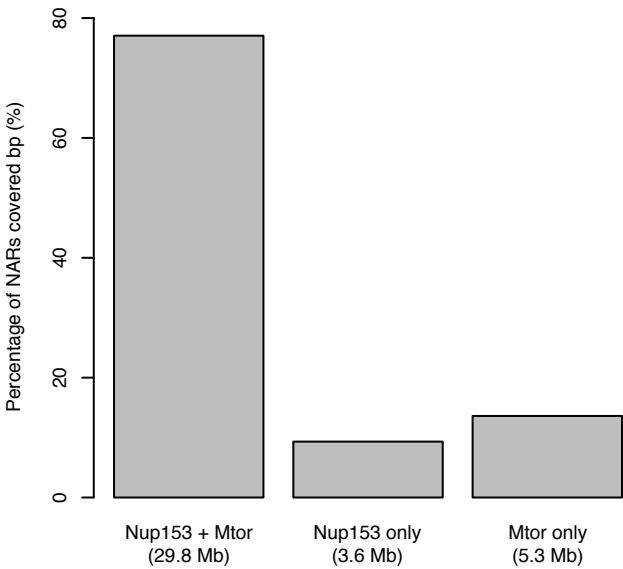

**C**

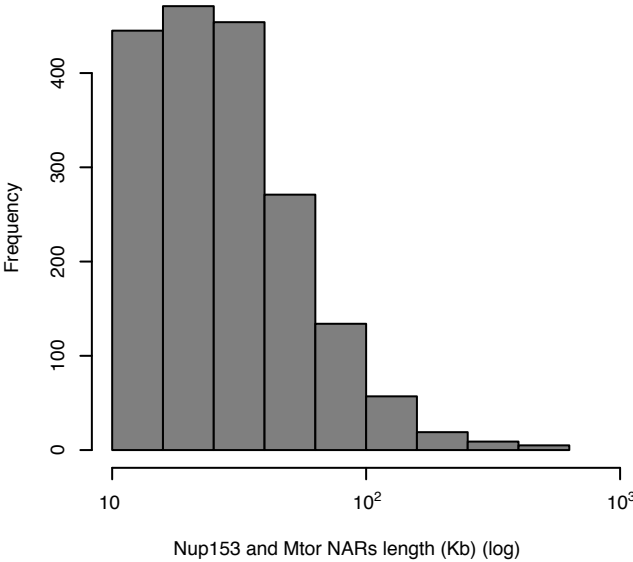

Supplement: Figure S4 — Correlation between Nup153 and Mtor binding in Kc cells. (A) Smoothed scatter plot displaying the ChIP/input binding ratios for Nup153 and Mtor (Pearson r = 0.88). (B) Bar chart representing the overlap in NARs defined by Nup153 and Mtor binding profiles. (C) Histogram of Nup153 and Mtor NAR length distributions. (0.56 MB PDF) [file pgen.1000846.s004.pdf]

# Supplementary Figure 5

A

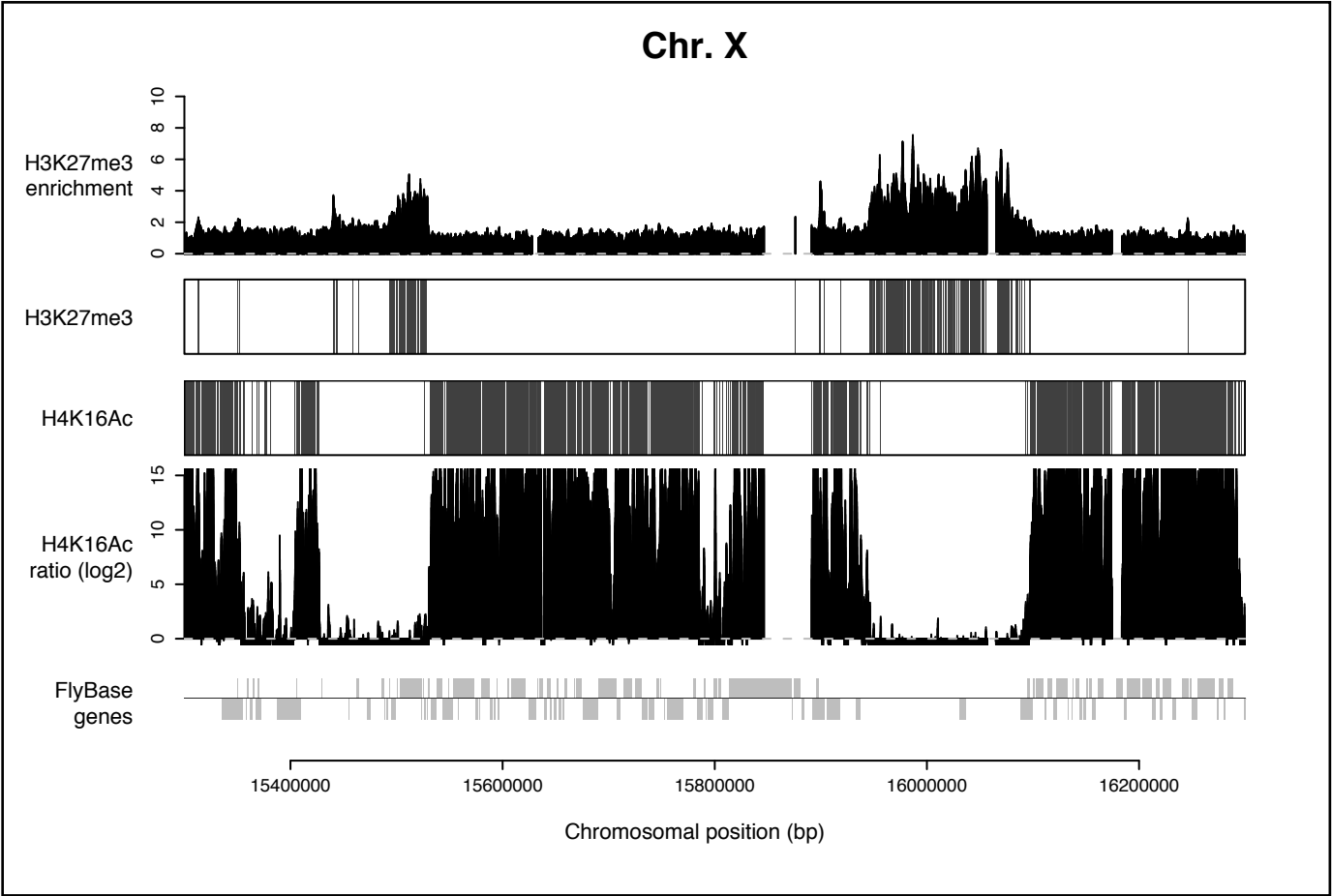

B

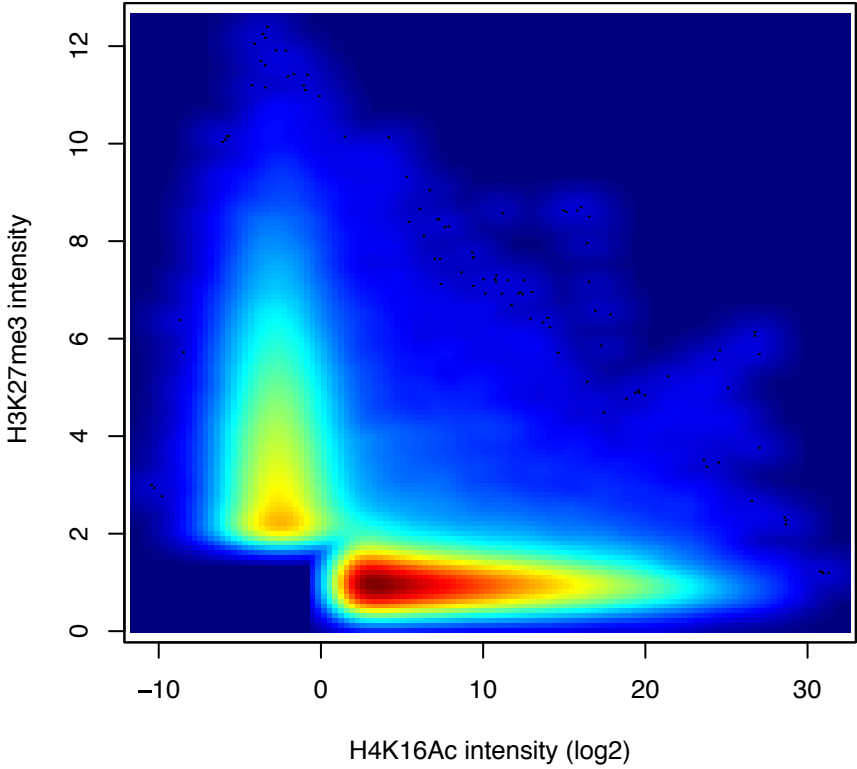

Supplement: Figure S5 — H4K16Ac and H3K27me3 are mutually exclusive throughout the genome. (A) Detail view of H3K27me3 and H4K16Ac modifications in a 1 Mb region of chromosome X in SL-2 cells. H3K27me3 data were obtained from Schwartz et al (2006) [36] and H4K16Ac data were obtained from Kind et al (2008) [32]. For each modification, we used the cut-offs from the original publications to define significant signals. (B) Smoothed scatter plot of H4K16Ac and H3K27me3 modification intensity values. Only data points with significant intensity values are shown. Plot areas with high data density are shown in dark red; plot areas with low are density are shown in dark blue. (1.39 MB PDF) [file pgen.1000846.s005.pdf]

Supplementary Figure 6

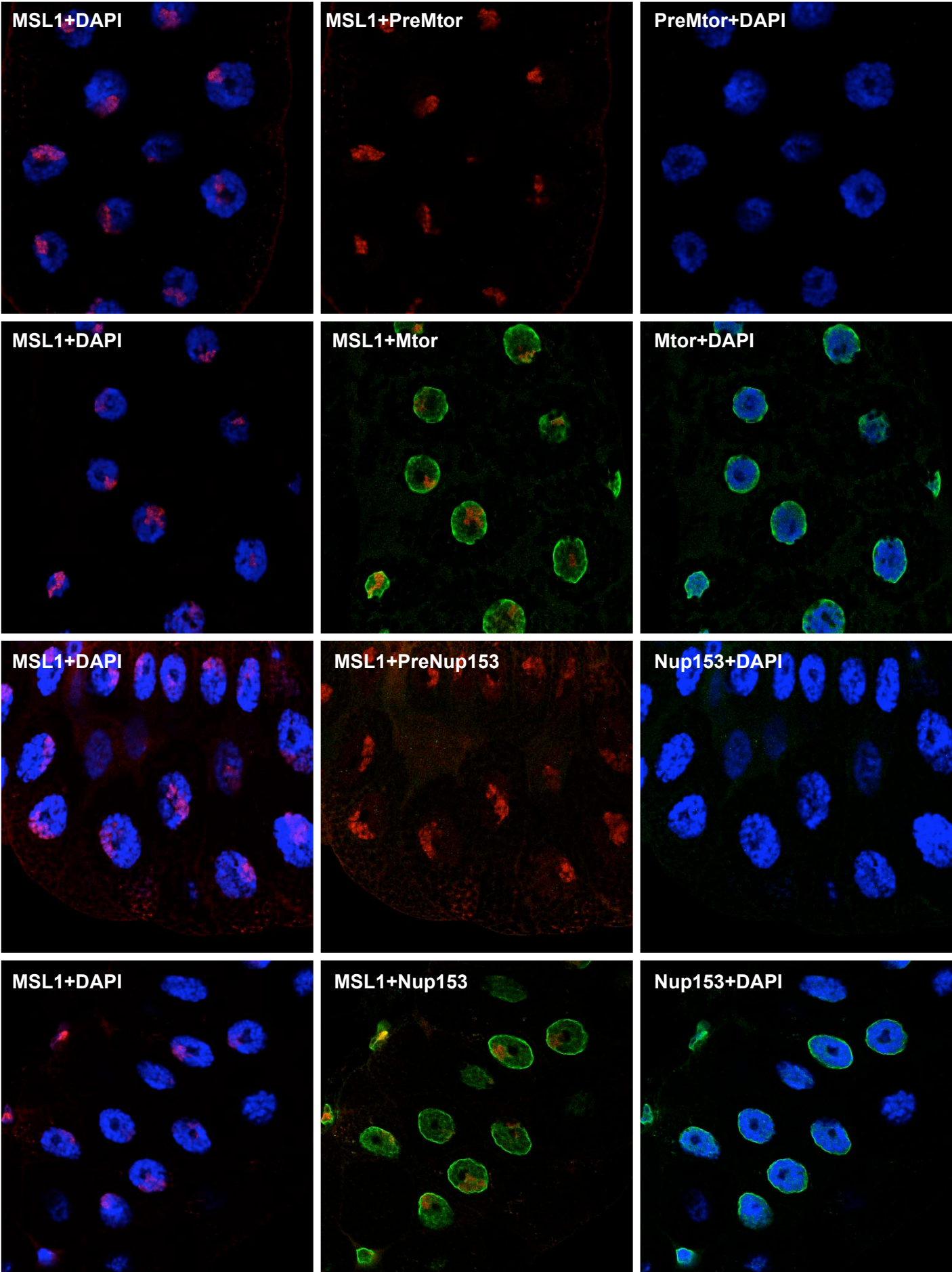

Supplement: Figure S6 — Immunostaining of Nup153 and Mtor in salivary glands. Immunostaining of Nup153 and Mtor in salivary glands isolated from 3rd instar male larvae. Salivary glands were co-immunostained with either MSL1 antibody or pre-immune serum (Pre-Mtor, Pre-Nup153) and serum (Mtor and Nup153). Both Nup153 and Mtor show predominantly nuclear rim staining but there is also some diffuse staining within the nucleus. X chromosomal territory is observed with MSL1 staining. (0.23 MB PDF) [file pgen.1000846.s006.pdf]

Supplementary Figure 7

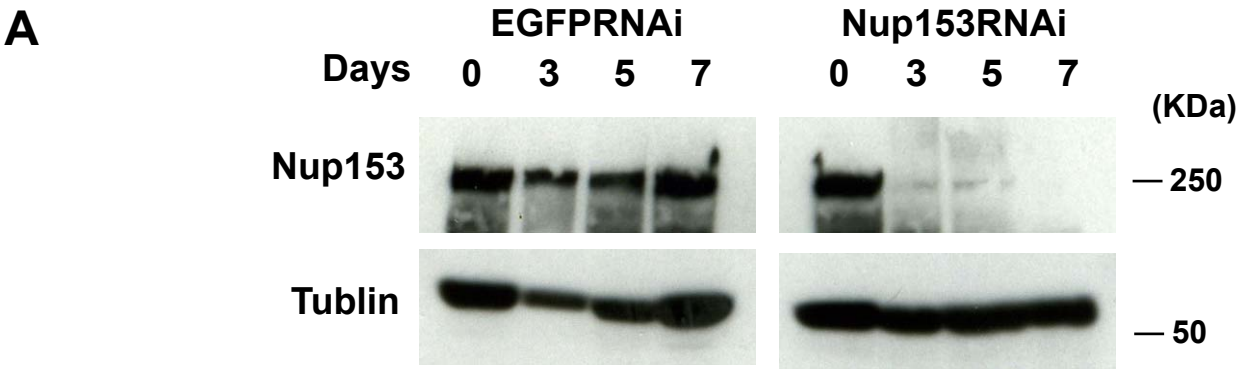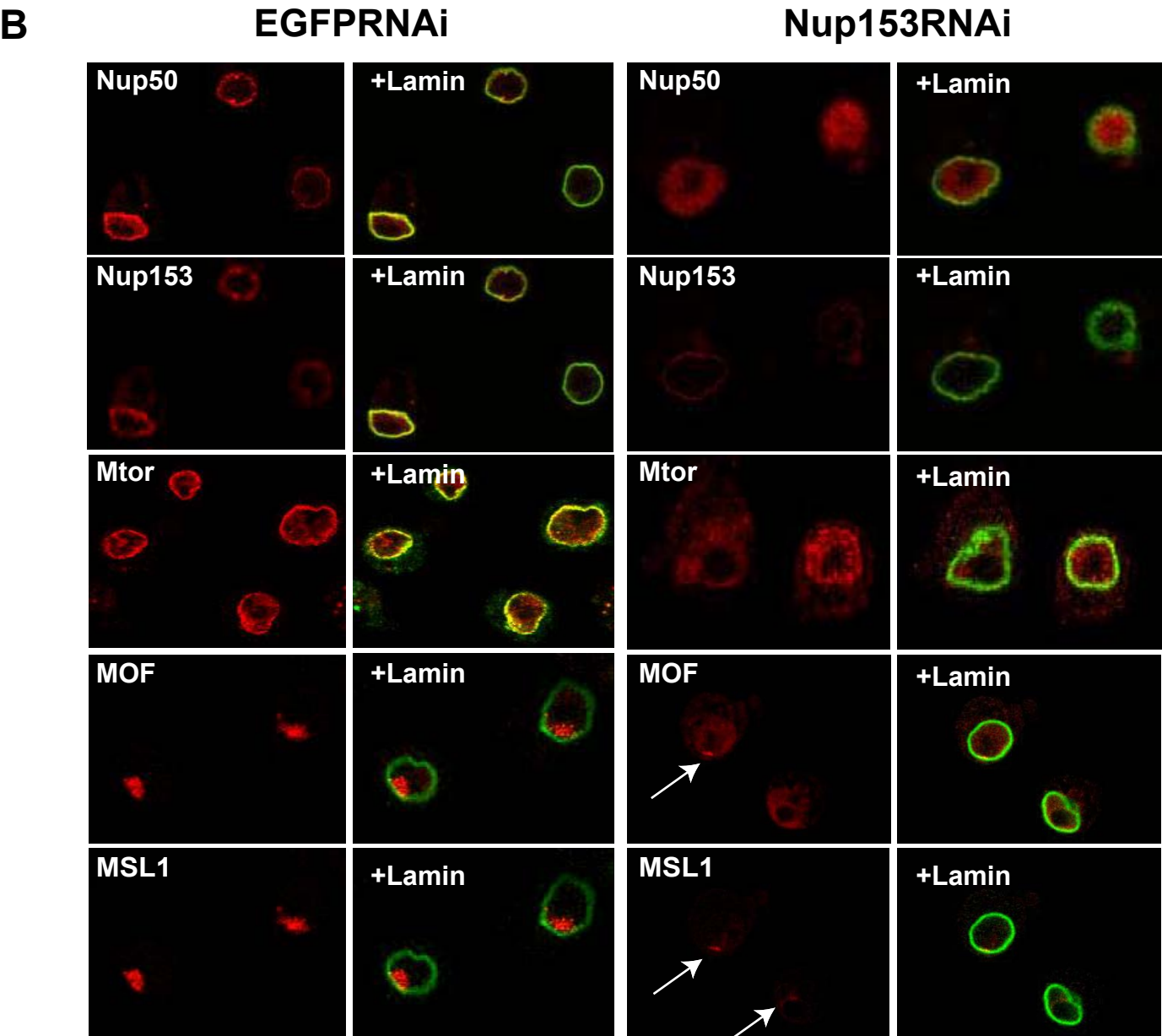

Supplement: Figure S7 — RNAi-mediated depletion of Nup153 in SL-2 cells. (A) Whole extracts were obtained from cells treated with EGFP or Nup153 dsRNA for 0, 3, 5, or 7 days, and separated on SDS PAGE followed by western blot analysis using Nup153 and Tubulin antibodies. Size markers (kDa) are indicated on the right side. (B) Cells treated with EGFP or Nup153 dsRNA were used for immunofluorescence confocal microscopy. Nup153, Nup50, and Lamin antibodies were used for triple-immunostaining and pseudo colours were added using the ImageJ software. A similar strategy was used for MOF, MSL1 and Lamin triple immunostaining. Arrows indicate residual MSL1- or MOF-staining in Nup153-depleted cells. (0.75 MB PDF) [file pgen.1000846.s007.pdf]

Supplementary Figure 8

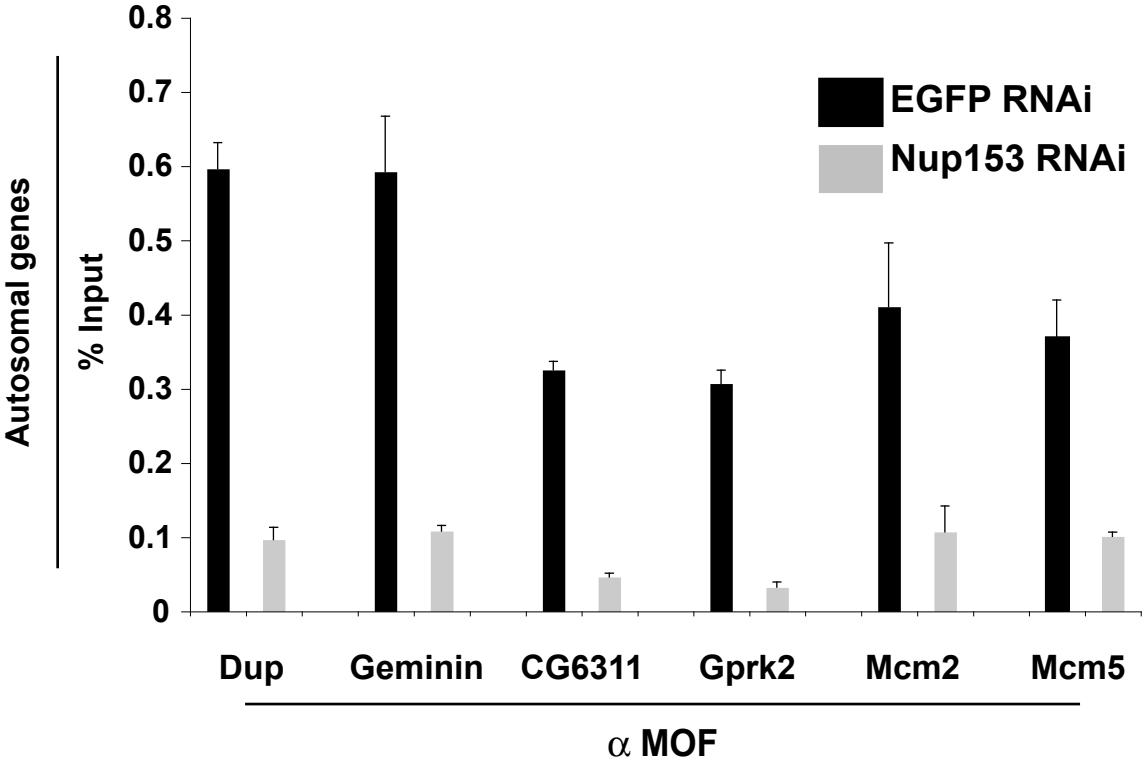

Supplement: Figure S8 — MOF-binding to autosomal promoters is affected in Nup153-depleted cells. Chromatin prepared from cells treated with EGFP (black) or Nup153 (grey) dsRNA was used for immunoprecipitation using MOF antibody. MOF-binding was scored on six autosomal target promoters. Recovered DNA was analysed by qPCR and is shown as percentage of input DNA (% Input). Error bars represent standard deviations obtained from three independent experiments. (0.33 MB PDF) [file pgen.1000846.s008.pdf]

Supplementary Figure 9

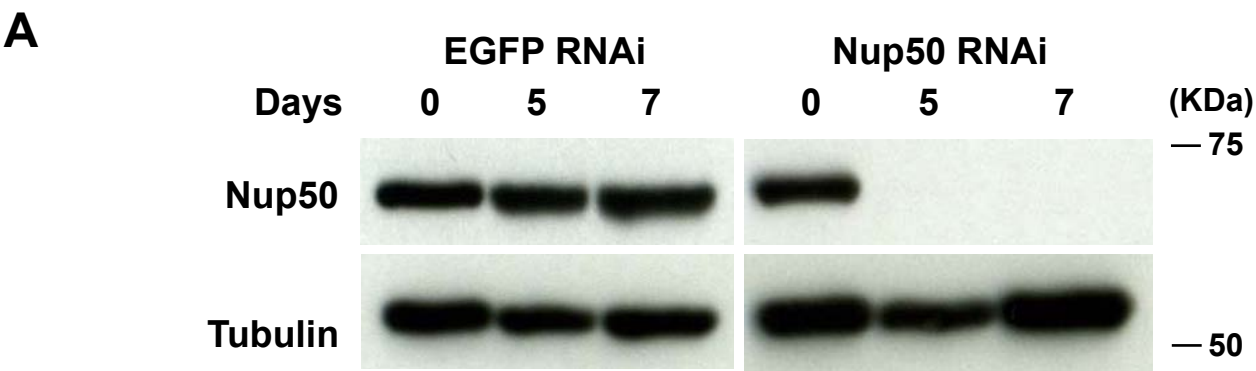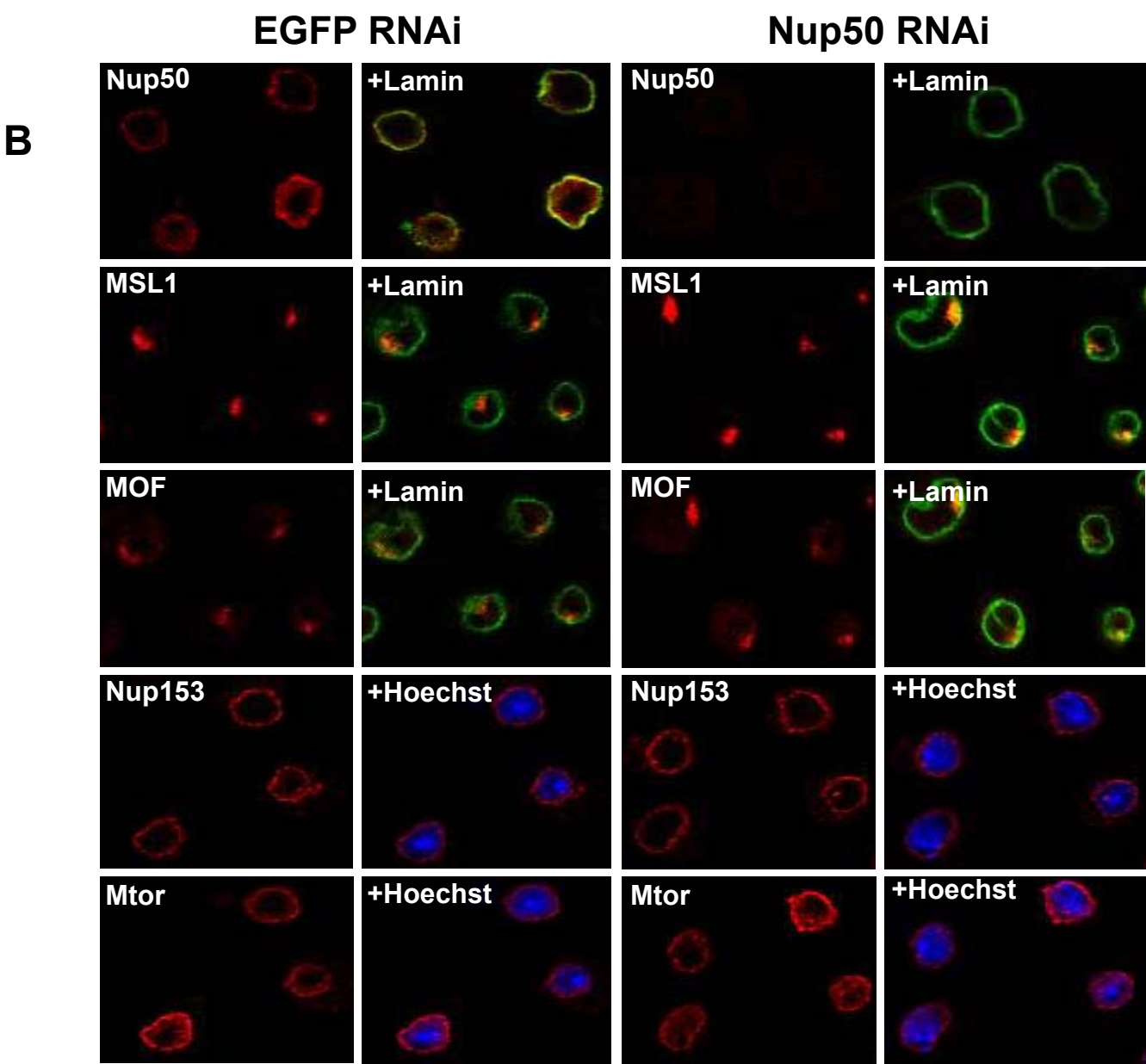

Supplement: Figure S9 — Control RNAi-mediated depletion of Nup50 in SL-2 cells. (A) Whole cells extracts were made from cells treated with EGFP or Nup50 dsRNA for 0, 5, or 7 days, and separated on SDS PAGE followed by western blot analysis using Nup50 and Tubulin antibodies. Size markers (kDa) are indicated on the right side. (B) Cells treated with EGFP or Nup153 dsRNA were used for immunofluorescence confocal microscopy using antibodies against Nup153, Nup50, Mtor, MOF, and MSL1 (shown in red). Nup153, Mtor antibodies, and Hoechst were used for triple-immunostaining and pseudo-colours were added using the ImageJ software. A similar strategy was used for MOF, MSL1, and Lamin triple immunostaining. (0.43 MB PDF) [file pgen.1000846.s009.pdf]

Supplementary Figure 10

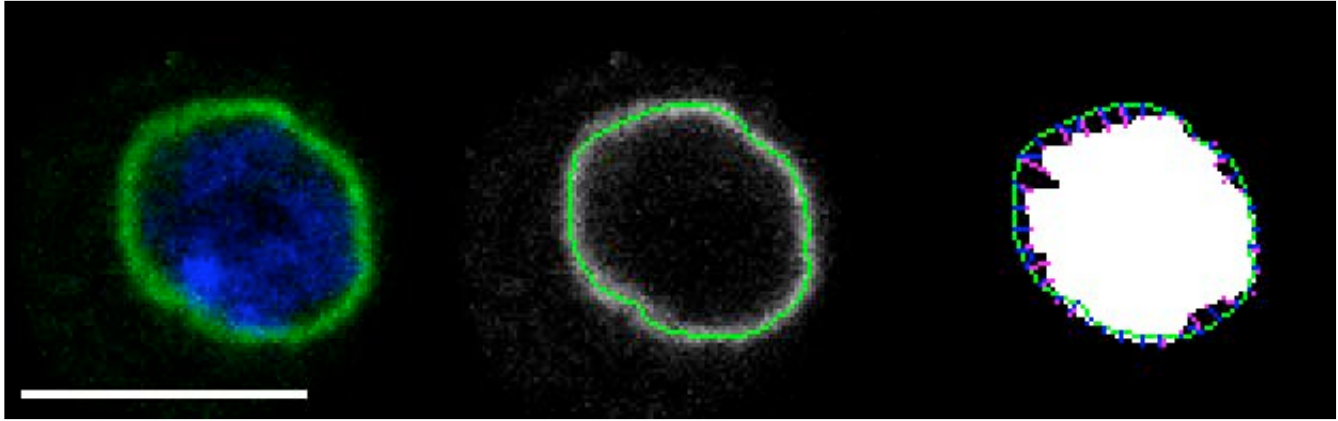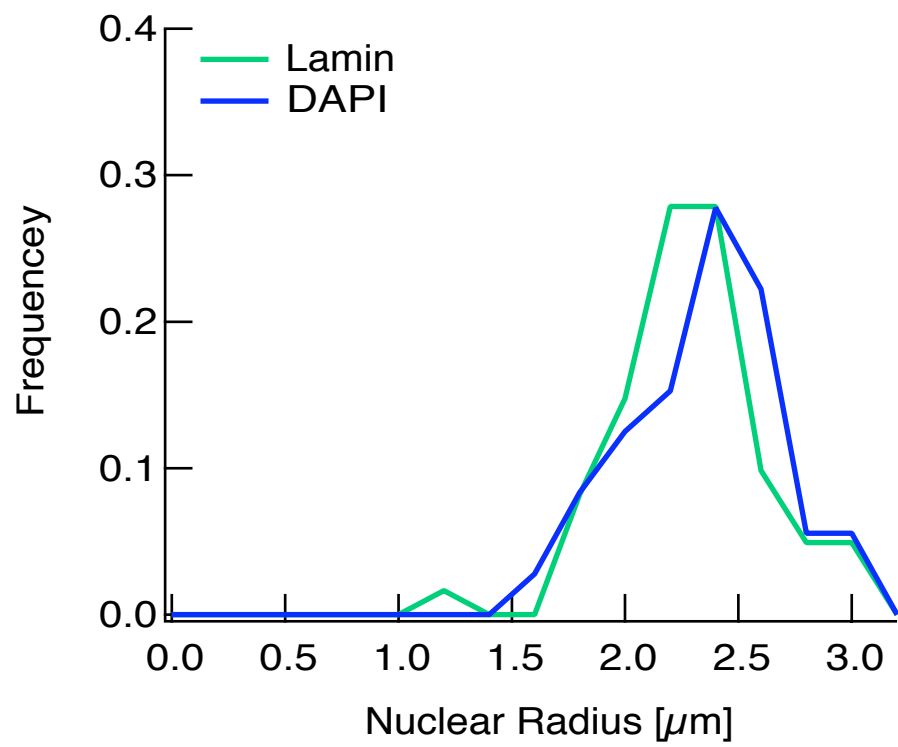

Supplement: Figure S10 — Segmentation of DAPI image. (Top left) Nucleus labelled with lamin and stained with DAPI were used for the development of segmentation method (green = lamin, blue = DAPI). (Top centre) Lamin signal was thresholded and then reduced to single pixel rim (green). Detected rim was overlaid to the original lamin image. (Top right) The segmentation strategy were verified by measuring the deviation between DAPI segmented image (black/white) and the lamin rim (green). Pink lines show how these deviations were measured. (Bottom) Probability density distributions of the mean radii of 62 individual nuclei calculated using the DAPI or lamin signals. The median radius for the DAPI segmented edge was 2.45±0.33 µm and for the lamin signal was 2.41±0.35 µm. (0.05 MB PDF) [file pgen.1000846.s010.pdf]

Supplementary Figure 11

A

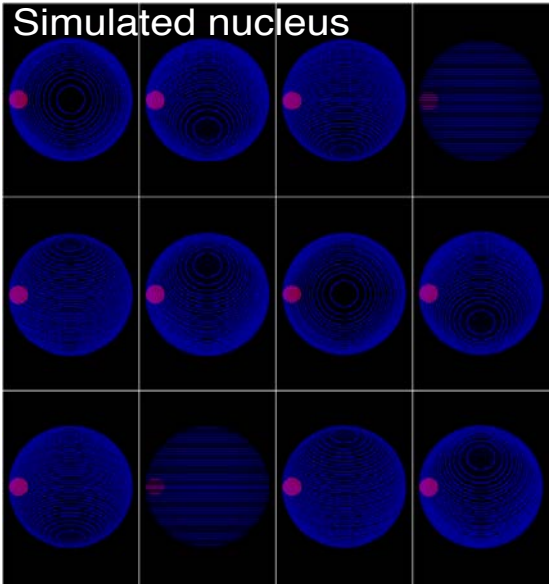

B

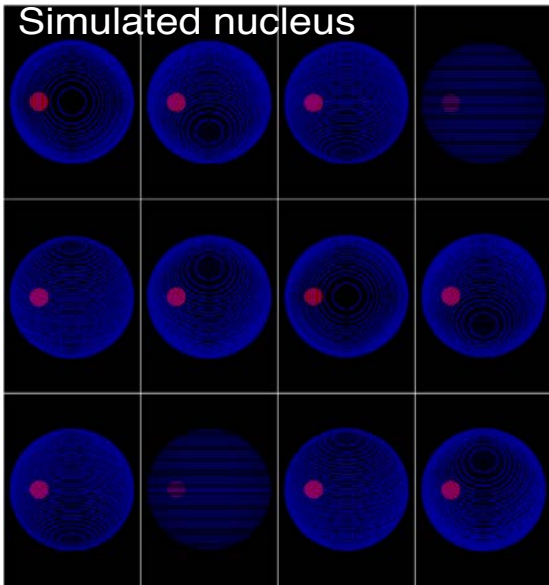

C

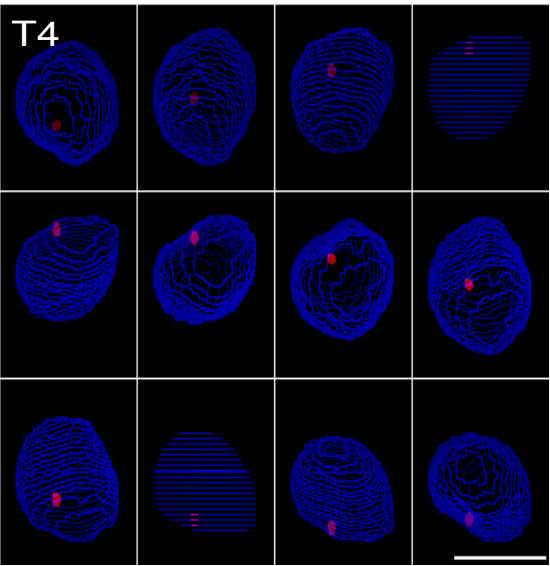

D

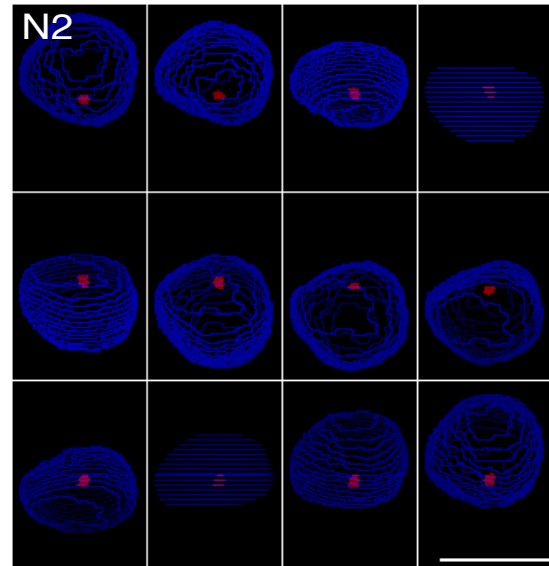

Supplement: Figure S11 — Measurement of three-dimensional distance of FISH signals from the nuclear periphery. Three-dimensional brightest-point projection images of a simulated nucleus showing (A) peripheral localisation and (B) non-peripheral localisation. Outlines of nuclear periphery in each z-slice (blue contours, DAPI channel) and FISH signal (red, FISH channel) are shown. Nucleus is rotated on the x-axis with 30 degree increments from top-left to bottom-right panel. Three-dimensional brightest-point projection images of real nuclei with NAR locus (C) T4 and (D) control locus N2. Bar = 5 µm. See also Videos S1-S4. (0.55 MB PDF) [file pgen.1000846.s011.pdf]
