# Supplementary material for: Nuclear Pore Proteins Nup153 and Megator Define Transcriptionally Active Regions in the Drosophila Genome
Source: PLoS Genet. 2010 Feb 12;6(2):e1000846. doi: 10.1371/journal.pgen.1000846 (PMC2820533; doi:10.1371/journal.pgen.1000846)
Supplement: Table S1 — Enrichment of active and repressive markers in NARs and non-NARs in SL-2 and Kc cells. (0.05 MB PDF) [file pgen.1000846.s012.pdf]

Supplementary Table 1

	NAR		Non-NAR		
	**# affected in NAR (% of total NAR)**	**Total # in NAR**	**# affected in non-NAR (% of total non-NAR)**	**Total # in non-NAR**	**NAR v non-NAR enrichment p-value**
SL-2 cells					
MOF binding (probes bound)	26,511 (3.3%)	815,694	17,140 (0.8%)	2,171,461	< 2.2e-16
RNA Pol II binding (genes bound)	2,530 (54%)	4,711	2,771 (30%)	9,328	< 2.2e-16
Active genes	2,870 (61%)	4,711	3,608 (39%)	9,328	< 2.2e-16
Nup153 RNAi DR genes*	1,067 (29%)	3,689	1,954 (19%)	10,350	< 2.2e-16
Lam binding (bp bound)	539,302 (1.7%)	31,941,190	1,981,179 (2.3%)	86,416,409	< 2.2e-16
H3K27me3 (probes modified)	44,043 (6.1%)	718,745	150,441 (7.8%)	1,929,043	< 2.2e-16
Kc cells					
MOF binding (probes bound)	18,813 (1.9%)	1,003,131	11,483 (0.58%)	1,984,024	< 2.2e-16
Active genes	3,772 (58%)	6,470	2,447 (32%)	7,569	< 2.2e-16
Nup153 RNAi DR genes*	934 (16%)	5,861	798 (9.8%)	8,178	< 2.2e-16

Enrichment of active and repressive markers in NARs and non-NARs in SL-2 and Kc cells.
*Total number of genes in NARs and non-NARs calculated for Nup153 only.
