# Supplementary material for: Nuclear Pore Proteins Nup153 and Megator Define Transcriptionally Active Regions in the Drosophila Genome
Source: PLoS Genet. 2010 Feb 12;6(2):e1000846. doi: 10.1371/journal.pgen.1000846 (PMC2820533; doi:10.1371/journal.pgen.1000846)
Supplement: Table S2 — Enrichment of H4K16Ac and gene density in NARs versus non-NARs for SL-2 and Kc cells. (0.05 MB PDF) [file pgen.1000846.s013.pdf]

Supplementary Table 2

	NAR		Non-NAR		
	**Mean value**	**1st quantile / 3rd quantile**	**Mean value**	**1st quantile /3rd quantile**	**NAR v non-NAR comparison p-value**
SL-2 cells					
H4K16Ac (intensity signal; log2)	2.374	-1.888 / 5.342	-1.86	-3.073 / 0.003	< 2.2e-16
Gene density (in 20 Kb window)	3.557	1 / 5	2.834	1 / 4	< 2.2e-16
Kc cells					
H4K16Ac (intensity signal; log2)	1.761	-1.697 / 4.498	-0.826	-2.906 / -0.078	< 2.2e-16
Gene density (in 20 Kb window)	3.895	2 / 6	2.609	1 / 4	< 2.2e-16

Enrichment of H4K16Ac and gene density in NARs versus non-NARs for SL-2 and Kc cells.
