# Supplementary material for: Nuclear Pore Proteins Nup153 and Megator Define Transcriptionally Active Regions in the Drosophila Genome
Source: PLoS Genet. 2010 Feb 12;6(2):e1000846. doi: 10.1371/journal.pgen.1000846 (PMC2820533; doi:10.1371/journal.pgen.1000846)
Supplement: Table S3 — Target (T) and non-target (N) regions used for FISH analysis. Start and end show the chromosomal localization coordinates according to release 3 of the Drosophila melanogaster genome (R5.11). Genes in each probe set are also indicated. Individual genes within these regions, which were further tested by Q-PCR in this study, are indicated in red. (0.08 MB PDF) [file pgen.1000846.s014.pdf]

Supplementary Table 3

Target regions (T)				
**Probe ID**	**chromosome**	**Localization (R5.11) Start**	**End**	**genes in probe[a]**
T1	X	13493140	13509419	cg7107<br>cg11178<br>ndc80<br>BthD<br>tth
T2	3L	17519012	17534991	cg6311<br>cg7555
T3	X	19444436	19463999	cg3917(Grip84)<br>cg12230(car)
T4	X	2679659	2700714	cg32795<br>white
T5	X	1351062	1363179	cg14786<br>cg14787<br>cg14788(l(1)G0431)<br>o-fut2<br>cg32808<br>cg14777<br>cg14778
T6	X	19525092	19545830	e(y)3<br>18D10<br>Cg14213<br>cg12237<br>Sec61gamma<br>Arp11<br>Ranbp21
T7	X	5757550	5777882	cg15764<br>cg3033<br>mof<br>cg3016<br>cg16721
T8	X	7389851	7406897	cg32719
T9	2R	10789452	10813892	cg10249
T10	X	10109152	10125194	cg2962<br>cg15308<br>cg2967(cg34408)<br>cg32689<br>cg2961(Ipod)

T11	X	9453030	9478098	cg32702<br>cg32699
T12	2L	10963884	10987416	cg33129<br>YL-1<br>Cg16743<br>abo<br>l(2)06225<br>SCAR<br>Piwi
T13	3R	19499276	19520496	cg13822<br>cg10157<br>eIF-3p66<br>cg16710<br>cg18754<br>SPE<br>cg10254
T14	3R	2908960	2935835	Cg1091<br>alphaTub84B<br>cg4612<br>Alh
T15	X	11470931	11499247	cg11695<br>Rox2<br>Nod<br>Cg1561<br>Rho-4<br>cg2533
T16	3R	1184672	1203560	cg16708<br>cg31542<br>cg34277<br>RpII18<br>Hd<br>cg14677<br>7B2
T17	2L	6984984	6969921	snRNP70K<br>SP1070
T18	3L	1759322	1778277	Dhc62B<br>cg13933<br>cg12018<br>cg2021<br>mRpL46
L105	X	1082652	1097047	cg1380<br>cg14625
**Non-Target regions (N)**				
**Probe ID**	**chromosome**	**Localization (R5.11)**<br>**Start**	**End**	**genes in probe**
N1	3R	8491135	8501699	cg6188<br>cg14395

N2	3L	634019	653308	cg12030<br>cg3402
N3	2L	387860	403507	cg4213
N4	X	19898916	19921965	cg17004(D2R)
N5	X	19854428	19876863	cg9565(nep3)
N6	2L	728926	748130	Hsp60B<br>Eaat2
N7	3R	10524575	10545517	cg33967
N8	2L	423967	448326	BBS8<br>ex

Target (T) and non-target (N) regions used for FISH analysis. Start and end show the chromosomal localization coordinates according to release 3 of the Drosophila melanogaster genome (R5.11). Genes in each probe set are also indicated. [a] individual genes with in these regions which were further tested by Q-PCR in this study are indicated in red.
