# Supplementary material for: Nuclear Pore Proteins Nup153 and Megator Define Transcriptionally Active Regions in the Drosophila Genome
Source: PLoS Genet. 2010 Feb 12;6(2):e1000846. doi: 10.1371/journal.pgen.1000846 (PMC2820533; doi:10.1371/journal.pgen.1000846)
Supplement: Table S4 — This table accompanies Figure 4. Chromosomal location of the target and non-target regions is indicated. Total number of pixels and nuclei counted is also indicated as well as the statistical significance of each target or non-target region shown separately as well as average of each category. (0.06 MB PDF) [file pgen.1000846.s015.pdf]

Supplementary Table 4

Region	Location (chr. X or autosome)	Pixel points measured (n)	Number of nuclei measured	Wilcoxon p - value vs L105 (FDR adjusted)	Wilcoxon p-value vs N2 (FDR adjusted)
**Target**					
T2	A	10568	64	<0.0001	1.0000
T9	A	3188	60	0.0041	0.0423
T12	A	6370	70	0.0043	0.0984
T13	A	7325	70	0.1297	0.0047
T14	A	12800	65	<0.0001	0.5378
T16	A	1353	35	<0.0001	0.5602
T17	A	2833	72	0.0015	0.3276
T18	A	4520	84	0.0015	0.1634
T1	X	8335	68	0.0028	0.2023
T3	X	5834	55	0.0041	0.2409
T4	X	7451	76	0.5995	0.0006
T5	X	3940	90	0.4691	0.0013
T6	X	3493	44	0.0547	0.0690
T7	X	9115	54	0.3564	0.0013
T8	X	8101	83	0.7863	0.0002
T10	X	7787	55	0.0039	0.1460
T11	X	6717	61	0.0101	0.0984
T15	X	6476	66	0.2952	0.0013
**Non-Target**					
N1	A	5239	51	<0.0001	0.7841
N2	A	10930	63	<0.0001	1.0000
N3	A	9810	91	<0.0001	0.3901
N6	A	7206	66	0.0001	0.7495
N7	A	5237	87	0.0001	0.3276
N8	A	6324	71	0.1297	0.0069
N4	X	5281	54	0.6384	0.0047
N5	X	4507	57	0.1947	0.0069
**Cumulative distributions**					
Auto. targets		48957	520	<0.0001	0.0677
Chr. X targets		67249	652	0.0094	0.0003
Auto. non-targets		47063	429	<0.0001	0.2136
Chr. X non-targets		9581	111	0.2248	0.0005

This table accompanies Figure 4. Chromosomal location of the target and non-target regions is indicated. Total number of pixels and nuclei counted is also indicated as well as the statistical significance of each target or non-target region shown separately as well as average of each category.
