# Supplementary material for: Nuclear Pore Proteins Nup153 and Megator Define Transcriptionally Active Regions in the Drosophila Genome
Source: PLoS Genet. 2010 Feb 12;6(2):e1000846. doi: 10.1371/journal.pgen.1000846 (PMC2820533; doi:10.1371/journal.pgen.1000846)
Supplement: Text S1 — Primer sequences for quantitative PCR; primer sequences for RNAi. (0.09 MB PDF) [file pgen.1000846.s016.pdf]

Supplementary Material and Methods

Primer sequences for quantitative PCR
(distances defined from ATG)

White
P1 -108
F: TTTCACACTTTCCCCTGCTT
R: GCTGTGCCAAAACTCCTCTC

P2 3714
F: GCCGATAGGTCAGATGTCGT
R: TCCAGCAGGATGACCTCTTT

P3 5404
F: CCCGAAGTCTTAGAGCCAGA
R: TTAGCTGCACATCGTCGAAC

CG32699
P1 -220
F: CCTCACTGTCCACGACTTCA
R: GCGATCTGAGTTTGCGGTAT

P2 5225
F: GCCCGGTTTGAACTTAATCA
R: TTAACTATGCCCAGCCCATC

P3 6638
F: TCCAGCTTGACTCCATTTCC
R: CACAAATCGGAGCACATACG

CG2967
P1 -309
F: CGCACCTGCACCTAAATTCT
R: GGGATCGTGGTAATCCTCCT

P2 2230
F: CATCGGGTGCTACGAACTCT
R: GAAAGAGAAGCAGCGTGTCC

P3 8800
F: TTAGCTACCAACCGCCTCAT
R: CCTCGCTCAGATCCATTTTG

MOF
P1 -236
F: GTGCTTTCTCTGAAGGTTTTGG

R: GCAACCCTGATTTATCACAACA

P2 684
F: CAACATGACACGCTACCAGAAG
R: ACCAGGTGTCGATCTCGTAGTT

P3 1883
F: ACCAGGTGTCGATCTCGTAGTT
R: CTAGCATACGGAACCTGGAGAC

CG14788
P1 -108
F: TCGCAAATCCACAGAAGAAA
R: AGGAATCGAATCTCGCCATA

P2 681
F: AGTAGAAGGCCGTGCGAATA
R: CGCTACGTAAAGGAGGTGGA

P3 1885
F: TCCAGGTGCGAGAACTTTTT
R: CACGTCAAAGGACGCACTAA

dup
P1 -560
F: GTATTCGATCCGCTTGGAAA
R: CTTGCGAAGCCAGGTAAGTC

Geminin
P1 -220
F: ACACGAAATCGAGGGATGAA
R: CAACGCTTCACAGTTTTCCA

CG6311
P1 -600
F: AAACTAGCTGCGTGGCAAAT
R: GCACCCAGAAGCCTTAACAG

GprK2
P1 -110
F: CTTGTTTTGCGAGCCTTTTC
R: CAGAACACACACACGCACAC

Mcm2
P1 -185
F: GAAGCGGGTGTCCACTGTAT
R: GCTAATCGAGCCAACTCAGG

Mcm5
P1 -108
F: CAAAGCACGCAGCTGTAAAC
R: CATCTGCCACATCCATTTTG

CG6188
P1 -295
F: TAATCAAAACCGGAGGGTCA
R: CTCCCACATAGTGCCGACTT

P2 1181
F: TGACCAGTGAGTTCCGTCTG
R: ATGTAGAAGGCGGGGTTCTT

CG3402
P1 -239
F: TAAGATTGTCGCCCACAACA
R: TGCCAAACAGCAATCGTAAA

P2 1148
F: TAAGTGATGCGCCTTCTTCG
R: TACGTGCAGACCATTCCAAA

CG4213
P1 -611
F: AACGCAACTTTTCGGTTAGC
R: CGAGAGAAGCCAAATTCGTT

P2 2061
F: GTTGTCGTATCCGGTTTGCT
R: AACTTTGCAACAGCCACAGA

P3 4551
F: GACTACGACGAGGCATGTCA
R: GGGGGCTTCCTTCTGAATAG

CG9565
P1 -2475
F: GTCGGCCAAACTTAATCGAA
R: TTGCGCTCATTTTCTTTGTG

P2 1880
F: ATATCACCATTCCGCTGGAG
R: TTGTCAAAGTGATCCGTCCA

P3 4101

F: TCGTGTCCGAAAGAAGAAGG
R: CCCACTAGCCCACCATTTTA

hb
P1 -3437
F: CGTTAGACCAACACGCAGAG
R: AATTCCGCGAAAAAGAAAGC

P2 -415
F: AGTGCGTATGATTGCGATTG
R: ACACAACAGAAGCAGCACCA

P3 1813
F: ATCGACTCATCTGCCGAACT
R: TCTCCCGAGCAACTGAAAGT

CG10249
P1 -515
F: CGGGAGAAAAACTTCACGAG
R: GGTCGGCAAAAGAAAAACAA

P2 6495
F: GACAGGGATCCAGCTCCATA
R: GTGGAGAGCGATGCTGGTAT

P3 15368
F: CACCCGGTTATCCTAAAGATG
R: TATCAAGCAACCGACAATGC

Primer sequences for RNAi

Nup153
TTAATACGACTCACTATAGGGAGAGCGAACAGTAGTTACCCGTCGATCAAC
TTAATACGACTCACTATAGGGAGAGTTGCGGCAGGGTTAAATGCTTCCGGC

Nup50
TTAATACGACTCACTATAGGGAGAATCGGTCCGTCATAAAATTCC
TTAATACGACTCACTATAGGGAGATGAATGTTTCCGAGAGAGAAGG
